# Insights into triterpene synthesis and unsaturated fatty-acid accumulation provided by chromosomal-level genome analysis of *Akebia trifoliata* subsp. *australis*

**DOI:** 10.1038/s41438-020-00458-y

**Published:** 2021-02-01

**Authors:** Hui Huang, Juan Liang, Qi Tan, Linfeng Ou, Xiaolin Li, Caihong Zhong, Huilin Huang, Ian Max Møller, Xianjin Wu, Songquan Song

**Affiliations:** 1 Key Laboratory of Research and Utilization of Ethnomedicinal Plant Resources of Hunan Province, College of Biological and Food Engineering, Huaihua University, Huaihua, 418000 China; 2 Kunming Institute of Botany, Chinese Academy of Sciences, Kunming, 650201 China; 3 State Key Laboratory Breeding Base of Dao-di Herbs, National Resource Center for Chinese Materia Medica, China Academy of Chinese Medical Science, Beijing, 100700 China; 4 Department of Molecular Biology and Genetics, Aarhus University, Flakkebjerg, DK-4200 Slagelse, Denmark; 5 Institute of Botany, Chinese Academy of Sciences, Beijing, 100093 China

**Keywords:** Genomics, Lipids

## Abstract

*Akebia trifoliata* subsp. *australis* is a well-known medicinal and potential woody oil plant in China. The limited genetic information available for *A. trifoliata* subsp. *australis* has hindered its exploitation. Here, a high-quality chromosome-level genome sequence of *A. trifoliata* subsp. *australis* is reported. The *de novo* genome assembly of 682.14 Mb was generated with a scaffold N50 of 43.11 Mb. The genome includes 25,598 protein-coding genes, and 71.18% (485.55 Mb) of the assembled sequences were identified as repetitive sequences. An ongoing massive burst of long terminal repeat (LTR) insertions, which occurred ~1.0 million years ago, has contributed a large proportion of LTRs in the genome of *A. trifoliata* subsp. *australis*. Phylogenetic analysis shows that *A. trifoliata* subsp. *australis* is closely related to *Aquilegia coerulea* and forms a clade with *Papaver somniferum* and *Nelumbo nucifera*, which supports the well-established hypothesis of a close relationship between basal eudicot species. The expansion of *UDP-glucoronosyl* and *UDP-glucosyl transferase* gene families and *β-amyrin synthase-like* genes and the exclusive contraction of *terpene synthase* gene families may be responsible for the abundant oleanane-type triterpenoids in *A. trifoliata* subsp. *australis*. Furthermore, the *acyl-ACP desaturase* gene family, including 12 *stearoyl-acyl-carrier protein desaturase* (*SAD*) genes, has expanded exclusively. A combined transcriptome and fatty-acid analysis of seeds at five developmental stages revealed that homologs of SADs, acyl-lipid desaturase omega fatty acid desaturases (FADs), and oleosins were highly expressed, consistent with the rapid increase in the content of fatty acids, especially unsaturated fatty acids. The genomic sequences of *A. trifoliata* subsp. *australis* will be a valuable resource for comparative genomic analyses and molecular breeding.

## Introduction

*Akebia trifoliata* (Thumb.) Koidz. subsp. *australis* is a perennial woody plant that belongs to the genus *Akebia* (Lardizabalaceae) and is mainly distributed in East Asia^1^. *A. trifoliata* subsp. *australis* (abbreviated as *A. trifoliata* hereafter) as well as *A. trifoliata* subsp. *trifoliata* and *A. quinata*, which are two other members of the genus *Akebia*, are listed in the Chinese Pharmacopoeia^2^ and have been used as traditional herbal medicines for >2000 years. An example is “Akebiae Fructus”, which is made from the dry fruit of *A. trifoliata*, including seeds, peel, and flesh^3,4^. Experimental and clinical studies have demonstrated that *A. trifoliata* possesses anti-inflammatory, antimicrobial, antioxidative, and anticancer properties^4,5^. Lu et al.^5^ reported that the ethanol extract of *A. trifoliata* seeds has antimetastatic potency against hepatocellular carcinoma cells. The pharmacological properties of *A. trifoliata* are attributed to numerous bioactive compounds, including triterpenoid saponins, triterpenes, and flavonoids, in dried fruits, stems, leaves, and seeds of *A. trifoliata*^6^. The content of oleanane-type triterpenoids, especially oleanolic acid, is high in the dry fruit of *A. trifoliata*^7^. These natural bioactive compounds with minimal or no side effects have attracted attention from chemists and pharmacologists due to their complex structural features and multiple biological effects.

Studies have indicated that the upstream reactions of triterpenoid biosynthesis through the mevalonate (MVA) or methylerythritol phosphate (MEP) pathways are involved in making building blocks and intermediates such as isopentenylpyrophosphate and farnesyl diphosphate (FPP)^8^. The downstream reactions of triterpenoid biosynthesis start from the condensation of two molecules of FPP, and the triterpenoid backbone undergoes oxidation, substitution, and glycosylation to generate various triterpenoids, involving many key enzymes, including squalene synthase (SQS), squalene monoxidase, oxidosqualene cyclase (OSC), cytochrome P450 monooxygenase (CYP), and uridine diphosphate glycosyltransferase (UGT)^9^. Among these enzymes, *β*-amyrin synthase (*β*-AS), a kind of OSC, is a unique key enzyme that catalyzes oleanane-type triterpenoid biosynthesis, and its expression is positively correlated with oleanane-type saponin content^10^.

As a potential oilseed medicinal plant, *A. trifoliata* seeds contain up to 39% oil with 77% unsaturated fatty acids^3^. Owing to the high UFA content, the seed oil of *A. trifoliata* is used as a quality edible oil and dietary supplement in China. In contrast to saturated fatty acids (SFAs), the dietary potency of UFAs has health benefits by reducing the risk of cardiovascular disease, obesity, and cancer^11^ and promoting the absorption of lipophilic nutritional compounds^12^. Fatty acids (FAs) are synthesized in plastids from acetyl-CoA up to 18:1^Δ9^, the first desaturation catalyzed by stearoyl-acyl-carrier protein desaturase (SAD). After export to the cytosol, FAs can be elongated and desaturated to produce long-chain and polyunsaturated fatty acids (PUFAs) by various enzymes within the ER and eventually processed into the storage lipid triacylglycerol (TAG)^13^. TAG is stored in oil bodies that serve as a natural protective system against fatty-acid oxidation and maintain lipid stability^14^. In olive oil, monounsaturated oleic acid (C18:1) makes up 75% of all TAGs, followed by saturated palmitic acid (C16:0; ~13.5%), polyunsaturated linoleic acid (C18:2; ~5.5%), and α-linolenic acid (C18:3; ~0.75%)^15^. In sesame seed oil, both oleic acid and linoleic acid are more evenly present (~40%)^16^. The differential accumulation of oleic and linoleic acids in olive compared with sesame is attributed to the functional divergence of oil biosynthesis pathway genes, such as ω-6 *fatty-acid desaturase 2* (*FAD2*) and *SAD*, following duplication^17^. Oleosin, the most abundant oil body-associated protein, is a major determinant of oil body size^18^ and has been suggested to contribute to the stability of oil bodies and their synthesis and metabolism^19^. The heterologous expression of castor bean oleosin in *Arabidopsis* led to a 20% increase in the ricinoleic acid content in TAGs^20^. Moreover, coexpression of oleosins with other TAG biosynthesis genes increased the oil content^21^.

In addition to the usages mentioned above, the cultivated fruit of *A. trifoliata* is consumed as a delicacy due to its delicious taste and abundance of nutrients. *Akebia trifoliata* belongs to the genus *Akebia* (Lardizabalaceae) and, like *Aquilegia coerulea* and *Papaver somniferum*, is a member of the basal eudicots, which possess more diverse floral morphologies than the core eudicots and monocots. The identification and investigation of floral genes in basal eudicots can be used as an evolutionary link between core eudicots and grasses^22^.

Despite the considerable importance of *A. trifoliata*, genomic information for the species is limited, which has hindered its study and utilization. Here, we report the sequencing and assembly of a high-quality chromosomal-level genome sequence of *A. trifoliata* subsp. *australis*, which is the first sequenced species in the genus *Akebia*. Furthermore, we identified key genes involved in triterpene synthesis and the accumulation of UFAs. The availability of this genomic information will be helpful for comparative genomic analysis and the molecular breeding and engineering of *A. trifoliata*.

## Results and discussion

### *De novo* genome sequencing, assembly, and quality assessment

**Fig. 1:**
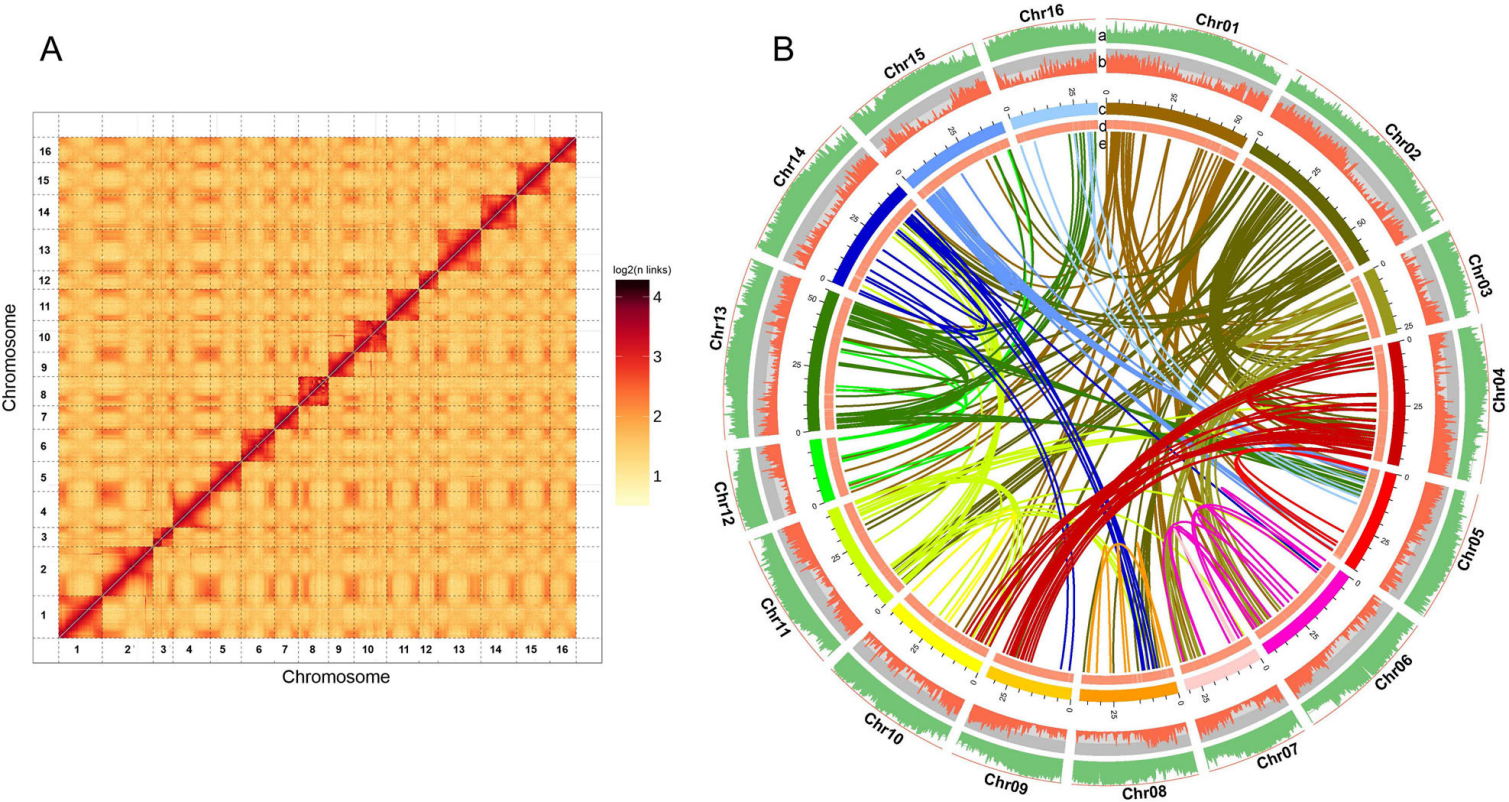
Chromosomal features of *Akebia trifoliata* subsp. *australis*. Hi-C map of the *A. trifoliata* subsp. *australis* genome showing genome-wide all-by-all interactions (**A**) and characterization of the *A. trifoliata* subsp. *australis* genome (**B**). **a** repeat density; **b** gene density; **c** chromosomes; **d** GC content; and **e** relationship between synteny blocks

The genome of *A. trifoliata* was initially sequenced and assembled using the HiSeq X Ten sequencing platform from Illumina and PacBio single-molecule real-time (SMRT) sequencing technology, and the assembled contigs were anchored to pseudochromosomes using the Hi-C technique. K-mer analysis revealed that the estimated genome size of *A. trifoliata* was 669.76 Mb with a heterozygosity of 0.89% (Fig. S1). Furthermore, the genome size of *A. trifoliata* was estimated to be 654.34 Mb using flow cytometry (Fig. S2). In total, 193.71 Gb of high-quality sequences with a depth of 284-fold of the *A. trifoliata* genome were used to assemble the genome (Table S1). To obtain further chromosomal information about *A. trifoliata*, the sequences were then scaffolded and corrected using optical mapping data, and the resulting scaffolds were clustered into 16 pseudochromosomes (2*n* = 32), accounting for 98.05% (668.89/682.14 Mb) of the genome (Fig. 1, Fig. S3). The final chromosome-scale genome was 682.14 Mb in length with 689 contigs (contig N50 = 6.20 Mb) and 109 scaffolds (scaffolds N50 = 43.11 Mb) (Table 1). The final assembled sequence 682.14 Mb for *A. trifoliata* was close to the calculated estimated size (669.76 Mb) and to the size estimated by flow cytometry (654.34 Mb). The assembled size was marginally larger than the estimated size, probably because of the relatively high content of repetitive sequences (71.18%).

**Table 1:** Statistics of *A. trifoliata* subsp. *australis* genome sequencing, assembly, and annotation

Genomic feature	*A. trifoliata* subsp. *australis*
Assembled genome size (Mb)	682.14
PacBio reads (Gb)	65.96 (96.7 X^a^)
Illumina reads (Gb)	24.75 (36.28 X)
Hi-C (Gb)	103.00 (151.03 X)
Total reads (Gb)	193.71 (284.02 X)
GC content (%)	35.02
Percentage of anchoring	98.05%
Number of contigs	689
Contigs N50 (kb)	6,198
Contigs N90 (kb)	1,563
Number of scaffolds	109
Scaffold N50 (kb)	43,106
Scaffold N90 (kb)	30,967
Longest sequence length (kb)	64,151
Total repetitive sequences (Mb)	485.55 (71.18%)
Total protein-coding genes	25,598
Annotated protein-coding genes	25,008
Average length per gene (kb)	6.47
Total exon length (kb)	41.34
Average exon length (kb)	1.62
Total intron length (kb)	124.39
Average intron length (kb)	4.86

^a^Sequence coverage

In the sequencing and assembly quality assessment, 98.56% of Illumina short-insert reads could be aligned back to the final assembly. Moreover, BUSCO analysis showed that 1518 (94%) of the 1614 orthologs from the Embryophyta database were completely captured in our assembly, and CEGMA analysis showed that the assembled genome completely recalled 424 (93%) of the 458 core eukaryotic genes (CEGs) and 186 (75%) of the 248 highly conserved CEGs (Fig. S4).

### Genome annotation

A total of 25,598 protein-coding genes were predicted in the *A. trifoliata* genome through a combination of *ab* initio prediction, homology search, and RNA-Seq prediction. Of all the predicted protein-coding genes, 24,814 (97%) were annotated based on homology search and RNA-Seq reads, with only 3.1% deriving solely from *ab* initio gene prediction, suggesting that the results of the protein-coding gene prediction were high quality (Fig. S5a). The *A. trifoliata* genome had an average gene length of 6,47 kb and average exon and intron lengths of 1,62 kb and 4,86 kb, respectively (Table 1). By similarity search, 25,008 protein-coding genes (98%) had functional annotations in public databases from several species, including *N. nucifera* (57.51%), *V. vinifera* (13.91%), *Theobroma cacao* (3.05%), and others (25.52%) (Fig. S5b, c and d, Tables S2 and S3). With regard to nonprotein-coding genes, we identified 431 tRNAs, 97 miRNAs, 222 rRNAs, 94 snRNAs, and 332 snoRNAs in our assembly (Table S4).

Repetitive sequences generally account for a substantial part of plant genomes and have a close relationship with genome size variation and functional adaptation^23^. A total of 485.55 Mb (71%) of the *A. trifoliata* genome was identified as repetitive sequences, which is the same as the proportion in *P. somniferum* (71%)^24^ and higher than that in *M. cordata* (63%)^25^, both of which are medicinal plants and basal eudicots. Approximately 87% of *A. trifoliata* repetitive sequences were classified as transposable elements (TEs) (Table S5). TEs occupy a significant fraction of many eukaryotic genomes and play an important role in the increase in genome size among angiosperms^26^. In our assembly, retrotransposon (Class I) and DNA transposon (Class II) TEs accounted for 72% and 7% of the genome, respectively. Among all TEs, long terminal repeats (LTRs) were the most abundant category of TEs, with 32% *gypsy* and 10% *copia*. Interestingly, the proportion of Penelope-like element/large retrotransposon derivative (LARD) elements in *A. trifoliata* was higher than that in most sequenced plant species, accounting for 21% of the genome. LARDs are considered to be nonautonomous elements and are the remnants of the deletion of autonomous LTR retrotransposons^27^. Recent lineage-specific radiation of LARDs (13% of the whole genome) in the pomegranate genome is responsible for fruit development, such as coloration, by affecting the expression of putative *UDP-glucose:flavonoid glucosyltransferase* (*UFGT*) and *MYB* genes^27^. The abundant LARD elements in the *A. trifoliata* genome might also have a close relationship with many important characteristics, including secondary metabolite accumulation, as in pomegranate.

### Gene family analysis and phylogenetic tree construction

**Fig. 2:**
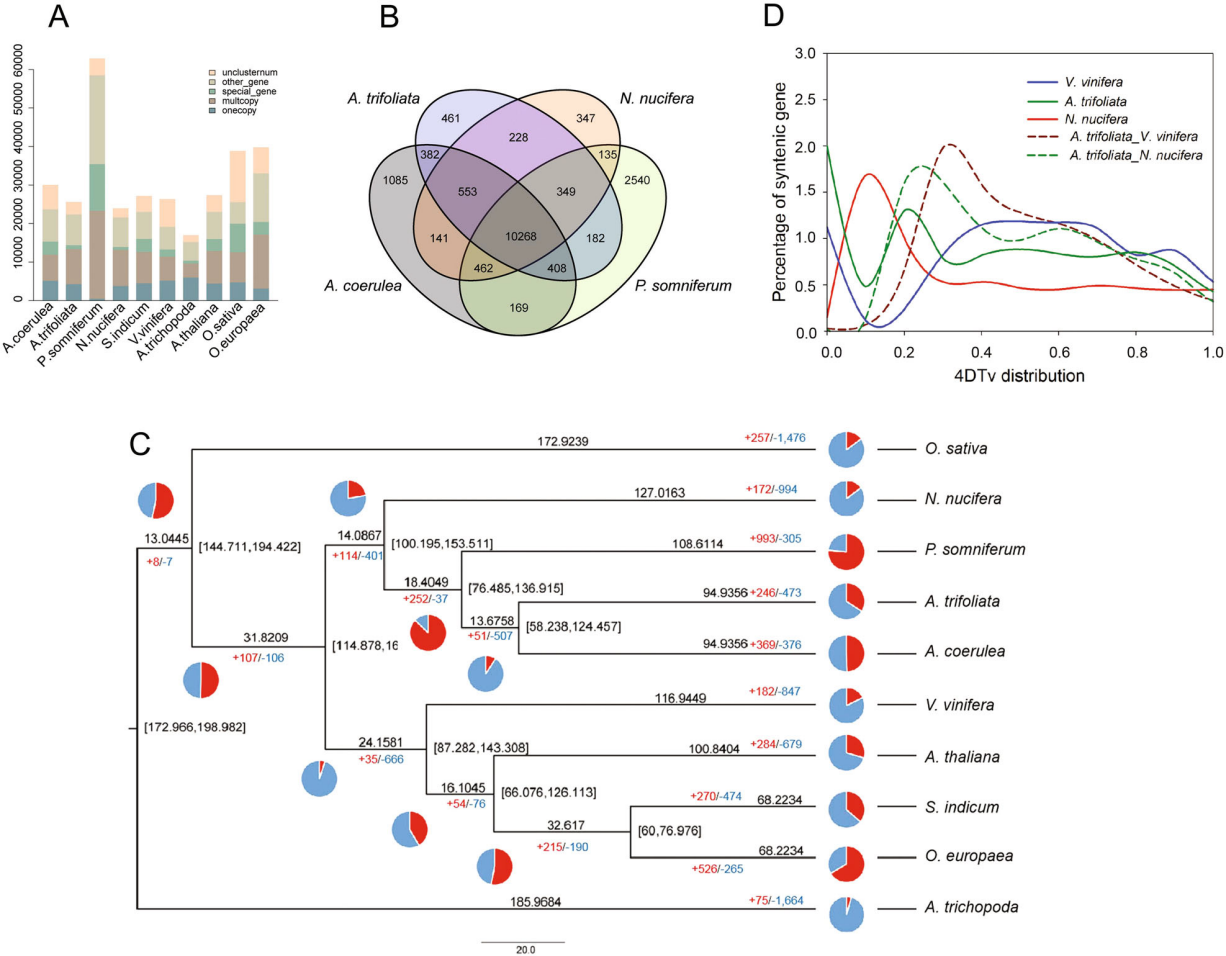
Comparative genomics analysis of *A. trifoliata* subsp. *australis* and other representative plant species. **A** Classification and statistics of common and lineage-specific genes in *A. trifoliata* and other representative plant species. **B** Venn diagram of gene families in *A. trifoliata* and three other basal eudicot species. **C** Phylogenetic analysis, gene family expansion/contraction analyses and divergence time estimations. The number of gene family contraction and expansion events are indicated by blue (the blue part of the pie chart) and red (the red part of the pie chart) numbers, respectively. Divergence times (million years ago, Mya) are indicated by the numbers beside the branch nodes. **D** Distribution of 4DTv among *A. trifoliata*, *N. nucifera*, and *V. vinifera* in intra- and intergenomic comparisons

We examined the evolutionary relationships among *A. trifoliata*, two eurosid species (*Arabidopsis thaliana*, *Vitis vinifera*), three basal eudicot species (*Aquilegia coerulea*, *Papaver somniferum*, and *Nelumbo nucifera*), two core eudicot oilseed species (*Sesamum indicum*, *Olea europaea*), a monocot species (*Oryza sativa*), and *Amborella trichopoda. A. trichopoda*, one of the basal angiosperm species, represents a sister group to other flowering plants^28^. By using gene family cluster analysis, we identified 12,831 gene families in *A. trifoliata*, of which 399 were unique gene families containing 1,028 genes (Fig. 2A, Table S6). Of those species*, P. somniferum* possessed the most genes (62,879) and unique gene families (2,394) owing to a relatively recent whole-genome duplication (WGD) event^24^. Then, we further compared the gene families among the four basal eudicot species. As shown in Fig. 2B, 10,268 gene families were shared by *A. coerulea*, *P. somniferum*, *N. nucifera*, and *A. trifoliata*. Compared with two other basal eudicot species, *P. somniferum* (2,662) and *A. coerulea* (1,085), *A. trifoliata* had fewer unique gene families (461). There were more shared gene family clusters between *A. trifoliata* and *A. coerulea* (11,611) than with any two of the other three species. Hence, we inferred a relatively close taxonomic relationship between the two species.

In total, 197 conserved single-copy orthologs were identified from the 10 species and were used to construct the phylogenetic tree with *O. sativa* and *A. trichopoda* serving as outgroups (Fig. 2C). Phylogenetic analysis revealed that *A. trifoliata* is closely related to *A. coerulea* and forms a clade with *P. somniferum* and *N. nucifera*, which supports the well-established hypothesis of a close relationship between basal eudicot species^25^. Based on the phylogenetic tree, we determined that 246 and 473 gene families were expanded and contracted, respectively, in *A. trifoliata* (Fig. 2C, Table S7). Gene ontology analysis also revealed that “supramolecular complex” and “cell-killing” appeared exclusively in expanded gene families, while “extracellular region part”, “cell proliferation”, and “biological adhesion” arose exclusively in contracted gene families (Fig. S6, Table S8).

### Divergence time estimation and whole-genome duplication analysis

Using MCMCtree, we estimated that the divergence between *A. trifoliata* and *A. coerulea*, which occurred ~94.94 Mya, whereas *P. somniferum* divergence occurred ~108.61 Mya (Fig. 2C). The previous estimates indicate that the divergence between *A. coerulea* and *M. cordata* occurred approximately 115.24 Mya^25^, suggesting a closer relationship between *A. coerulea* and *A. trifoliata* than between *A. coerulea* and *M. cordata*. The constructed tree provides the overall divergence time of important basal eudicot species. WGD has been found in almost all fundamental lineages of land plants and is considered a driver of diversity and adaptation^29^. The fourfold degenerate site transversion (4DTv) value revealed one peak at ~0.2 in *A. trifoliata*, which implies that it may have undergone WGD after divergence from *V. vinifera* and *N. nucifera* (Fig. 2D). An ancient paleohexaploidy (γ) event, occurring ~125 Mya, was detected in the common ancestor of all sequenced eudicot genomes^30^. According to the phylogenetic analysis and 4DTv values, we inferred that the γ event was absent in *A. trifoliata*, which is a basal eudicot species, as well as in the basal eudicot species lotus and *M. cordata*^25,31^. The WGD of basal eudicot species should be further analyzed.

### LTR insertion time

**Fig. 3:**
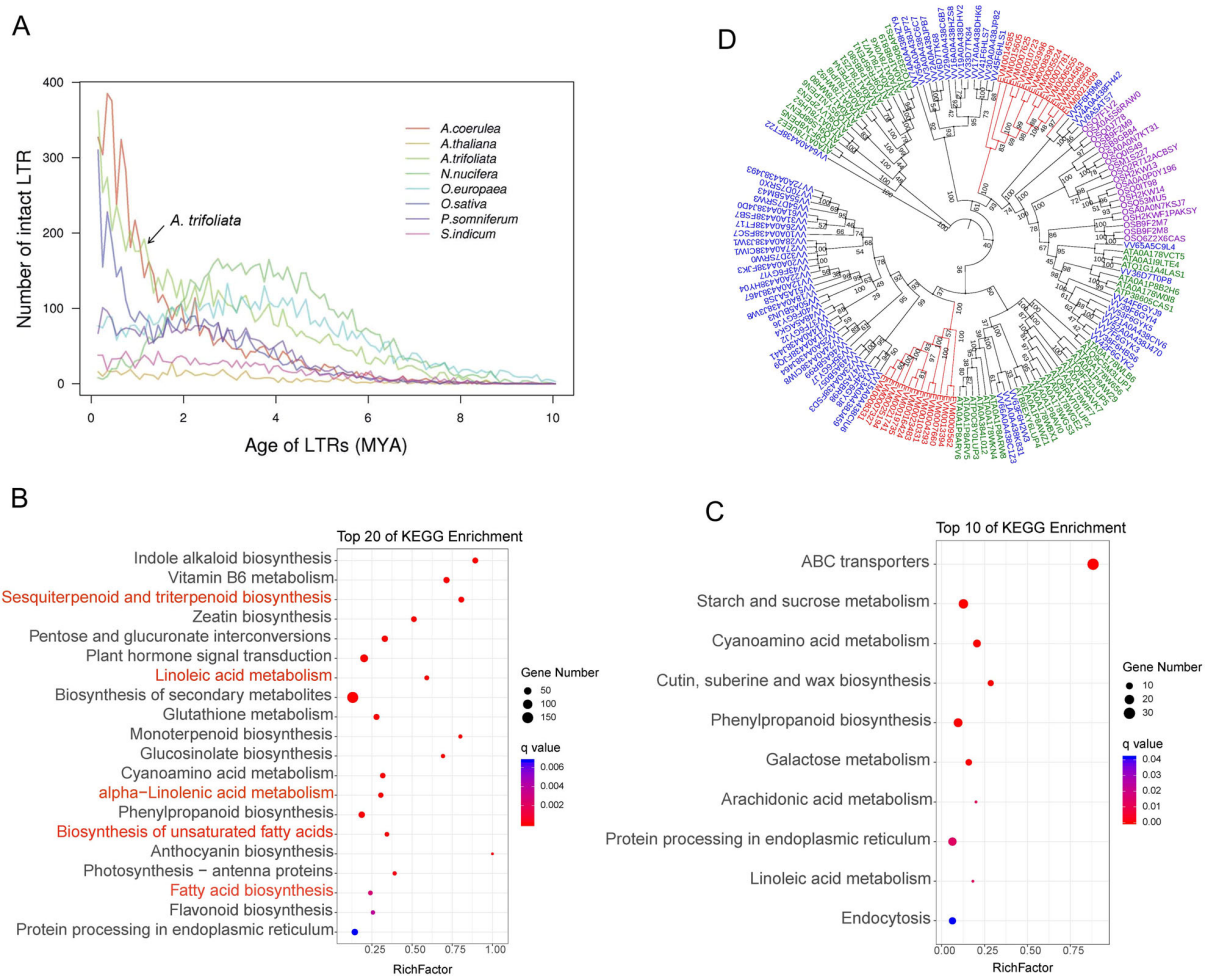
LTR insertion time estimation, enrichment analysis of expanded and contracted genes, and phylogenetic analysis of *β-amyrin synthase* genes. **A** LTR insertion time analysis in *A. trifoliata* and other representative plant species. **B**, **C** KEGG enrichment analysis of expanded and contracted genes in *A. trifoliata*. Red indicates pathways related to triterpenoid biosynthesis and fatty-acid metabolism. **D** Phylogenetic analysis of *β-amyrin synthase* genes in *A. trifoliata* (red), rice, *Arabidopsis* and grape. The protein sequences of *β-amyrin synthase* in *Arabidopsis*, rice and grape were downloaded from the UniProt database. The phylogenetic tree was constructed using the ML method with 1000 bootstrap replicates

LTR retrotransposons play an important role in genome instability and evolution, which affect the expression and profiles of nearby genes and have significant consequences for phenotypic variation^32^. To investigate the insertion time of LTRs in the *A. trifoliata* genome, we estimated the intrasequence divergence of identified full-length LTR elements. We discovered that massive recent insertion events of LTRs occurred in *A. trifoliata* within the last one million years and in *A. coerulea* and *O. sativa*, which explains the accumulation of many recent LTRs (Fig. 3A). The LTR amplifications were inferred to have taken place during the Pleistocene epoch in which freezing occurred and there was limited atmospheric CO_2_. Changes in the climate and environment cause serious survival stresses that force evolutionary adaptation by reorganization of genomes, represented by activated TEs^33^. Such an ongoing amplification of LTRs may have contributed to an especially large proportion of LTRs in *A. trifoliata*. Furthermore, the estimation of insertion times of LTRs suggests that *N. nucifera* and *O. europaea* experienced amplification events 2–5 million years earlier than *A. trifoliata*.

### Determination of functional genes involved in terpenoid biosynthesis

KEGG enrichment analysis indicated that the sesquiterpenoid and triterpenoid biosynthesis pathways and monoterpenoid biosynthesis pathway were significantly enriched in expanded genes (Fig. 3B, C). Notably, the 24 *β-amyrin synthase-like* (*Atrβ-AS*) genes involved in the sesquiterpenoid and triterpenoid biosynthesis pathways catalyzing the conversion of oxidosqualene to *β*-amyrin, the proposed aglycone of oleanane-type saponins^10^, were exclusively identified in the expanded gene set (Tables S7 and 8). Suppression of *β-AS* expression by RNA interference led to a reduced content of *β*-amyrin and oleanane-type saponins^10^. The systematic identification, prediction, and evolutionary analysis of *Atrβ-ASs* in *A. trifoliata* have been of great significance to our understanding of the synthesis and regulation of triterpene saponins. Phylogenetic analysis revealed that 24 *Atrβ-AS* genes were classified into two clusters, which formed two monophyletic groups. One *Atrβ-AS* gene (EMV0021809) was grouped together with three *Vitβ-AS* genes (Fig. 3D). The expansion of *Atrβ-AS* genes might be an explanation for the high content of hederasaponin and oleanolic acid in many tissues of *A. trifoliata*.

In the *A. trifoliata* genome, 12 UDP-glucoronosyl and 3 UDP-glucosyltransferase gene families were expanded and contracted, respectively, while 7 and 14 cytochrome P450 gene families were expanded and contracted, respectively. The downstream reactions in the biosynthesis of saponins are believed to include a set of cytochrome P450-dependent hydroxylation/oxidations and several glycosyl transfer reactions catalyzed by glycosyltransferases^34^. The frequent expansion and contraction of the two gene families indicate a rapid evolution of these gene families, which might be the cause of the great variety of triterpenes in *A. trifoliata*. Unexpectedly, terpene synthase (TPS) gene families (PF01397, PF03936), which are responsible for the synthesis of various terpene molecules, were contracted in the *A. trifoliata* genome (Table S7). We performed genome-wide identification of the TPS gene family and identified 34 *AtrTPS* genes (Table S9), which is much fewer than that of *V. vinifera* with 95 *TPS* genes. Moreover, the *TPS* genes of *A. trifoliata* and the other three species were used to construct a phylogenetic tree (Fig. S7), and they grouped into four distinct clusters. All *AtrTPS* genes were distributed in different branches of Cluster I, while Clusters II, III and IV comprised *TPS* genes of rice, *V. vinifera* and *Arabidopsis*, respectively. These results suggest that there is functional diversification among the *TPS* genes in those species and the *AtrTPS* genes in *A. trifoliata*. Evolutionary plasticity is evident in the TPS family, represented by different product profiles, subcellular locations, activities, and substrates.

### Molecular foundation for UFA accumulation

**Fig. 4:**
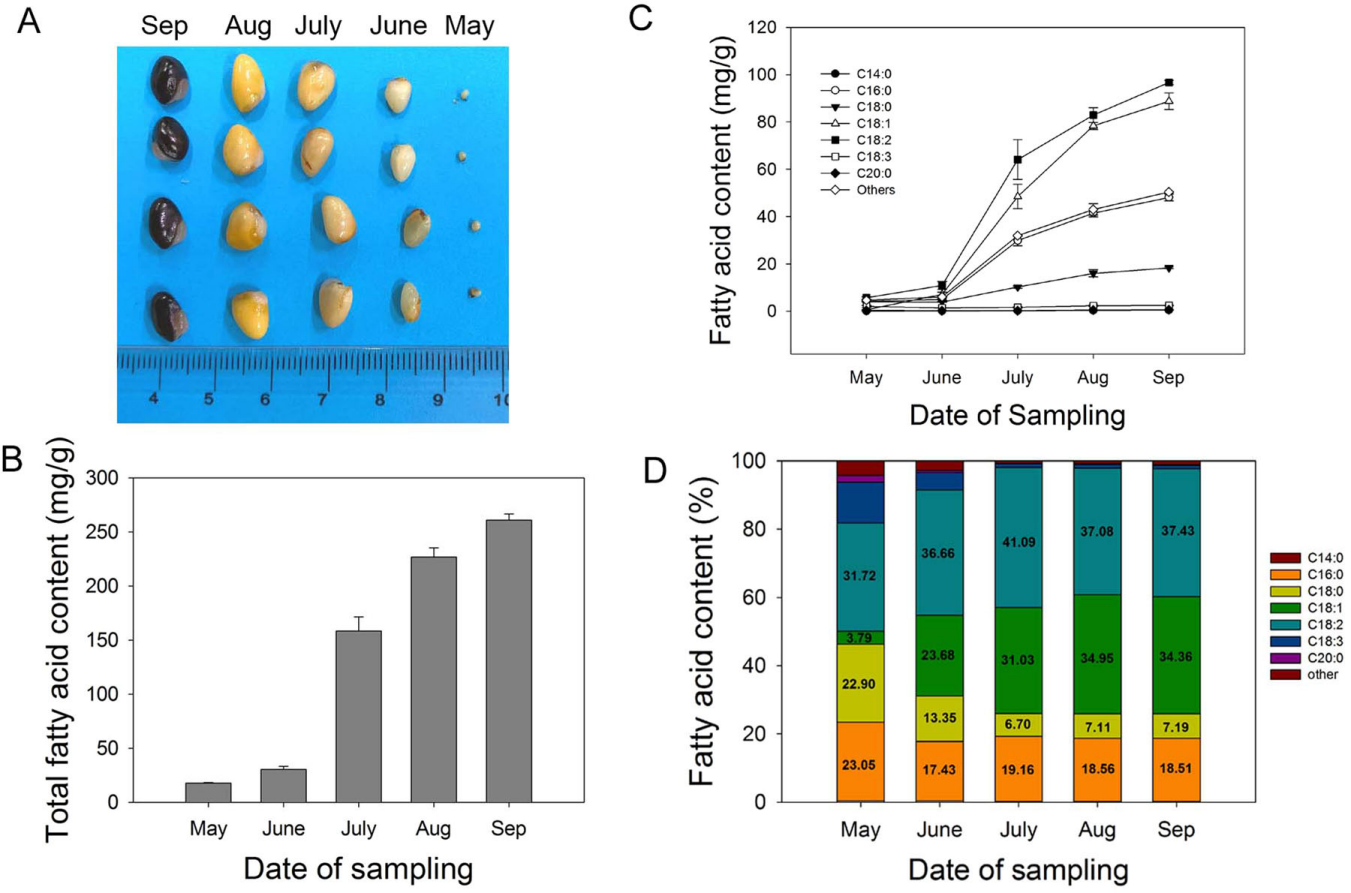
Changes in the seed content and percentage of total fatty acids (FAs) at different developmental stages. **A** Photos of seeds at different developmental stages. The scale bar is in cm. **B** Changes in the content of total FAs. **C** Changes in FA contents. **D** Changes in the percentage of FAs

The oil of *A. trifoliata* seeds has been suggested to have health benefits because of its high UFA content^3^. To further study FAs metabolism in *A. trifoliata* seeds, we determined the FAs composition and gene expression profiles at different stages of seed development (Fig. 4A). The total FA content increased 15-fold from May (17.8 mg g^−1^) to September (260.9 mg g^−1^), and FA accumulated dramatically from June (30.4 mg g^−1^) to July (158.3 mg g^−1^) (Fig. 4B). The time courses for the levels of the individual FAs were similar to that of the total FA content (Fig. 4C). A sharp rise (six- to sevenfold) in the contents of oleic acid, linoleic acid, and palmitic acid was also observed from June to July. The linolenic acid levels remained stable at ~2 mg g^−1^ during seed development. In May, linoleic acid (32%), palmitic acid (23%), and stearic acid (23%) were the dominant FAs in seeds, whereas oleic acid (3.8%) was much less abundant. With increasing seed maturity, we observed a significant increase in the proportion of oleic acid (34%) and linoleic acid (37%) and a reduced proportion of palmitic acid (19%), stearic acid (7%), and linolenic acid (0.95%) (Fig. 4D). In summary, the FA contents increased with seed development, and UFAs, including linoleic acid and oleic acid, accumulated especially rapidly during the early stages of seed development.

**Fig. 5:**
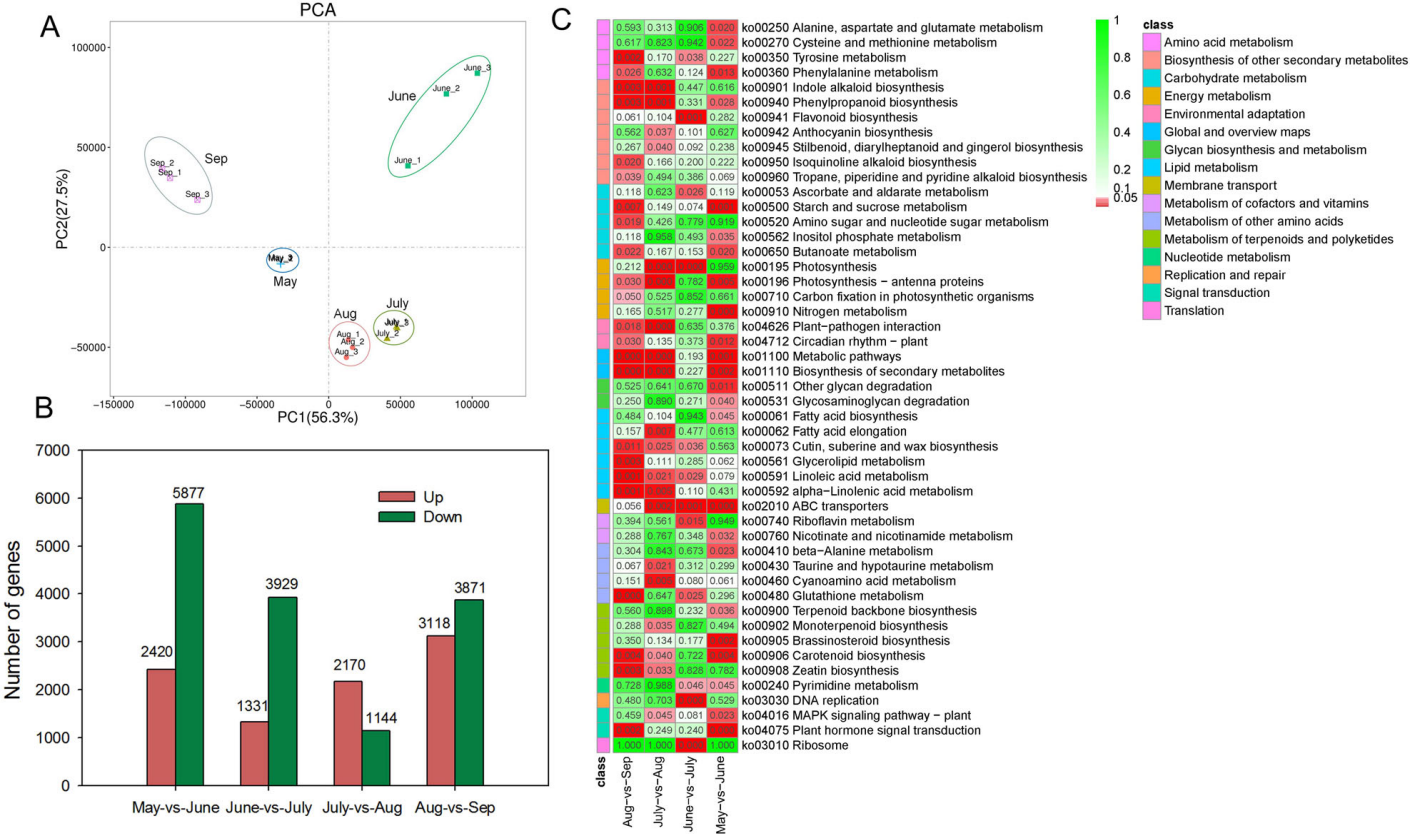
Transcriptomic analysis of seeds at different developmental stages. **A** PCA of different expression profiles in seeds. **B** Statistics on the numbers of differentially expressed genes (DEGs) in seeds at different developmental stages. **C** KEGG enrichment pathways in seeds at different developmental stages. The bar indicates the *p* values

**Fig. 6:**
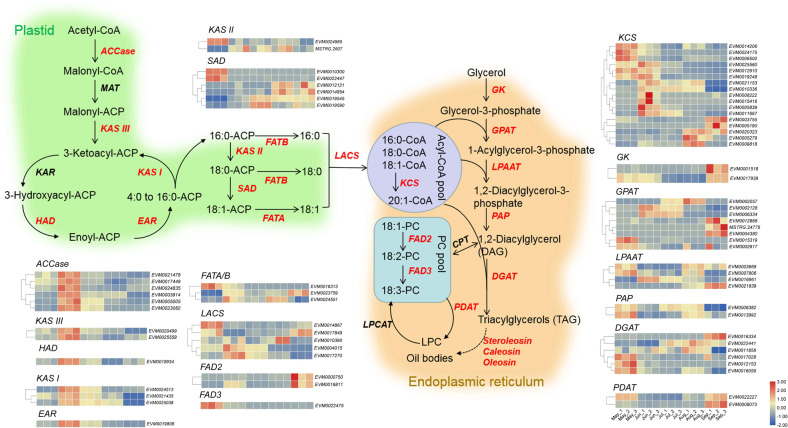
The expression levels of enzymes involved in FA synthesis and TAG assembly in developing *A. trifoliata* seeds. Gene expression levels in May, June, July, August, and September are indicated with colored bars (log2 FPKM). *ACCase* acetyl-CoA carboxylase, *CPT* diacylglycerol cholinephosphotransferase, *DGAT* acyl-CoA:diacylglycerol acyltransferase, *EAR* enoyl-acyl carrier protein (ACP) reductase, *FAD2* oleate desaturase, *FAD3* linoleate desaturase, *FATA/B* acyl-ACP thioesterase A/B, *GK* glycerol kinase, *GPAT* glycerol-3-phosphate acyltransferase, *HAD* hydroxyacyl-ACP dehydrase, *KAR* ketoacyl-ACP reductase, *KASI* ketoacyl-ACP synthase I, *KAS II* ketoacyl-ACP synthase II, *KAS III* ketoacyl-ACP synthase III, *KCS* 3-ketoacyl-CoA synthase, *LACS* long-chain acyl-CoA synthetase, *LPAAT* 1-acylglycerol-3-phosphate acyltransferase, *PAP* phosphatidate phosphatase, *LPCAT* 1-acylglycerol-3-phosphocholine acyltransferase, *MAT* malonyl-CoA:ACP malonyltransferase, *PDAT* phospholipid:diacylglycerol acyltransferase, *SAD* stearoyl-ACP desaturase. The colored boxes represent log_2_ FPKM values of DEGs in developing seeds, and sample names are shown on the lower right-hand side

Transcriptomic analysis revealed that the gene expression profiles of seeds in July and August were closer to each other and obviously differed from those of the three other stages (Fig. 5A). A total of 2420, 1331, 2170, and 3118 upregulated differentially expressed genes (DEGs) and 5877, 3929, 1144, and 3871 downregulated DEGs were identified compared with those of the previous month (Fig. 5B). There were more DEGs between May vs June and August vs September, which agrees with the principal component analysis results, demonstrating more marked changes in gene expression profiles at the early and later stages of seed development. KEGG enrichment analysis indicated that many DEGs were enriched in lipid metabolism pathways (Fig. 5C, Table S10). An expression heat map of these DEGs linked to FA metabolism, integrating the changes in FA contents during the development of *A. trifoliata* seeds, can provide valuable information about the regulation of oil accumulation and UFA synthesis. The reconstruction of the FA and TAG biosynthetic pathways includes *de novo* formation of acyl chains in the plastid and TAG assembly in the endoplasmic reticulum (Fig. 6). Almost all DEGs involved in the *biotin carboxyl carrier protein (BCCP) of acetyl-CoA carboxylase* (*ACCase*) and *fatty-acid synthase* (*FAS*) complexes including *β-ketoacyl-ACP synthase* (*KAS*), *β-hydroxacyl-ACP dehydratase* (*HAD*), and *enoyl-ACP reductase* (*EAR*), showed maximal transcription in June, followed by July. The transcription peak of the core FA biosynthetic machinery coincided with the onset of oil accumulation in the seed at the early developmental stage. *ACCase* catalyzes the conversion of acetyl-CoA into malonyl-CoA and is the rate-limiting enzyme in fatty-acid biosynthesis^35,36^. It has been proposed that lipid biosynthesis can be increased by overexpressing *ACCase*^37,38^. *KASI* catalyzes the elongation of *de novo* fatty acids, and *KASI* mutation results in a significant reduction in FA contents in seeds^39^. *EAR* catalyzes a key regulatory step in FA biosynthesis and shows the highest expression during the early stages of seed development^40^. In the oil palm mesocarp, the key FA biosynthesis genes were highly expressed at 120 days after pollination when oil accumulation began in the mesocarp^41^. We inferred that the significant upregulation of *ACCase* and *FAS* in seeds in June has a close relationship with the rapid increase in the oil content in July. Additionally, three *acyl-ACP thioesterases* (*FATA/B)* and three *long-chain acyl-CoA synthetases* (*LACS)* were also highly expressed in June. *FATA* and *FATB* play an essential role in chain termination during fatty-acid synthesis. LACS mediates FA transport and conversion to acyl-CoA^41^. However, many DEGs involved in triacylglycerol (TAG) biosynthesis, such as *glycerol kinase* (*GK*), *glycerol-3-phosphate acyltransferase* (*GPAT*), *1-acylglycerol-3-phosphate acyltransferase* (*LPAAT*), *phosphatidate phosphatase* (*PAP*), *acyl-CoA:diacylglycerol acyltransferase* (*DGAT*), and *phospholipid:diacylglycerol acyltransferase* (*PDAT*), were highly expressed throughout seed development.

**Fig. 7:**
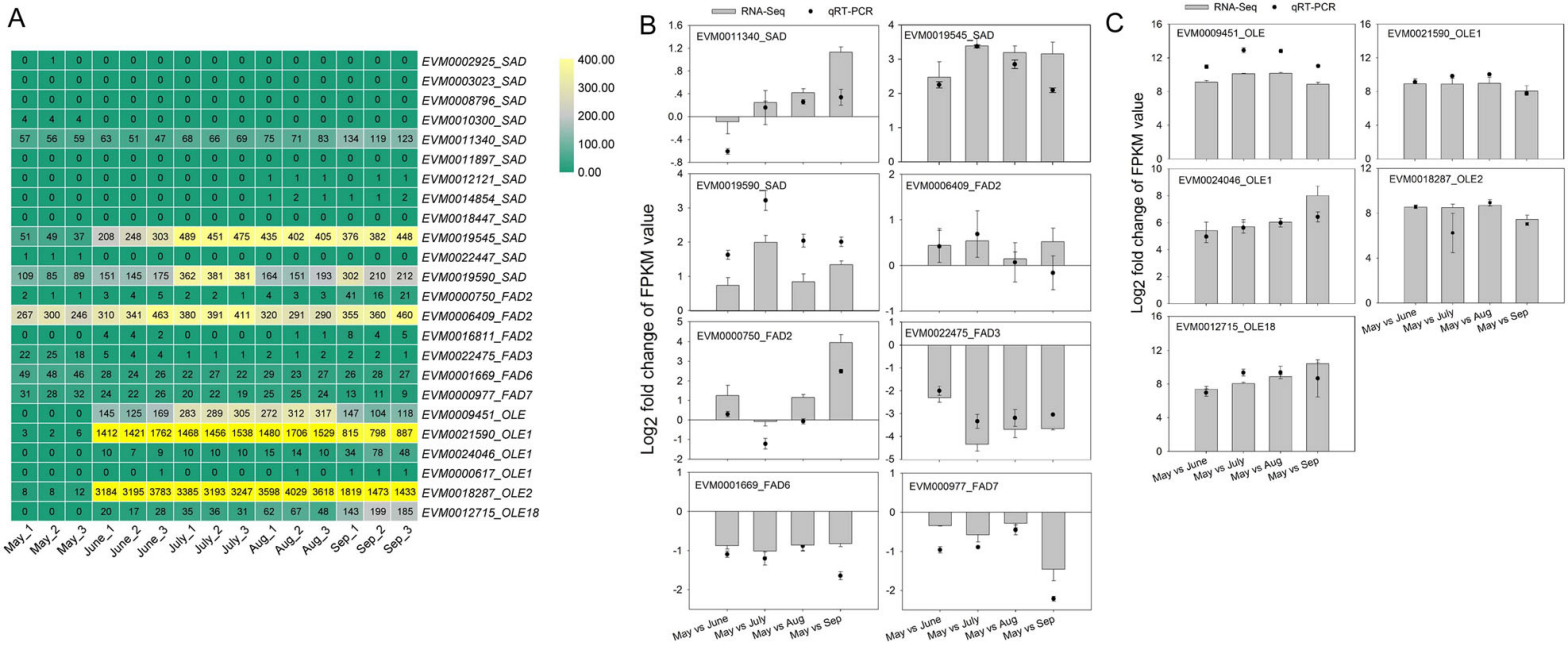
The expression levels of SADs, FADs, and oleosins in seeds at different developmental stages. **A** The log_2_ (FPKM) values. **B**, **C** The expression levels during seed development determined by RNA-Seq and qRT-PCR

Comparative genomics and enrichment analyses indicated that linoleic acid metabolism, α-linolenic acid metabolism, UFA, and FA biosynthesis were enriched in an expanded gene set (Fig. 3B). The expansion of those genes involved in lipid metabolism may be responsible for the high UFA and oil contents in *A. trifoliata* seeds. Among those expanded gene families, an acyl-ACP desaturase gene family (PF03405.9), including 12 *AtrSAD* genes involved in UFA biosynthesis, was expanded in the *A. trifoliata* genome. Furthermore, two *AtrSADs* (EVM0019545 and EVM0019590) were highly expressed during seed development and showed significantly increased expression levels in seeds in July (Fig. 7), which may contribute to the high oleic acid content in *A. trifoliata* seeds. Three omega *FAD* genes, *FAD2* (EVM0006409), *FAD6* (EVM0001669), and *FAD7* (EVM0000977), also remained at a high expression level during seed development (Fig. 7A, B), especially *FAD2. SAD* and *FAD2* play key roles in the synthesis of UFAs^42^. The suppression of *FAD2* expression by siRNA leads to a low linoleic acid content (5.5%) in olive oil^17^. It is noteworthy that homologs of oleosin (EVM0009451, EVM0021590, EVM0024046, EVM0018287, and EVM0012715), encoding oil body proteins that assist with packaging of TAG and determining oil body size, showed a sharp increase and a significantly higher expression in seeds from June to September compared with that in May (Fig. 7A, C). For example, the FPKM values of the two *AtrOLEs* (EVM0021590 and EVM0018287) in seeds were 441- and 366-fold higher in June than in May, respectively, which was confirmed by qRT-PCR (Fig. 7C). The results indicate that the significant upregulation of *ACCase* and *FAS* in FA biosynthesis, the high levels of *SAD* and *FAD2* in FA desaturation, and the stabilization of oil bodies by oleosin allow *A. trifoliata* to accumulate high levels of oil. In *Jatropha*, increased expression of FA biosynthesis genes and oleosins synergistically results in the accumulation of high levels of oil in kernels (~63%)^43^.

**Fig. 8:**
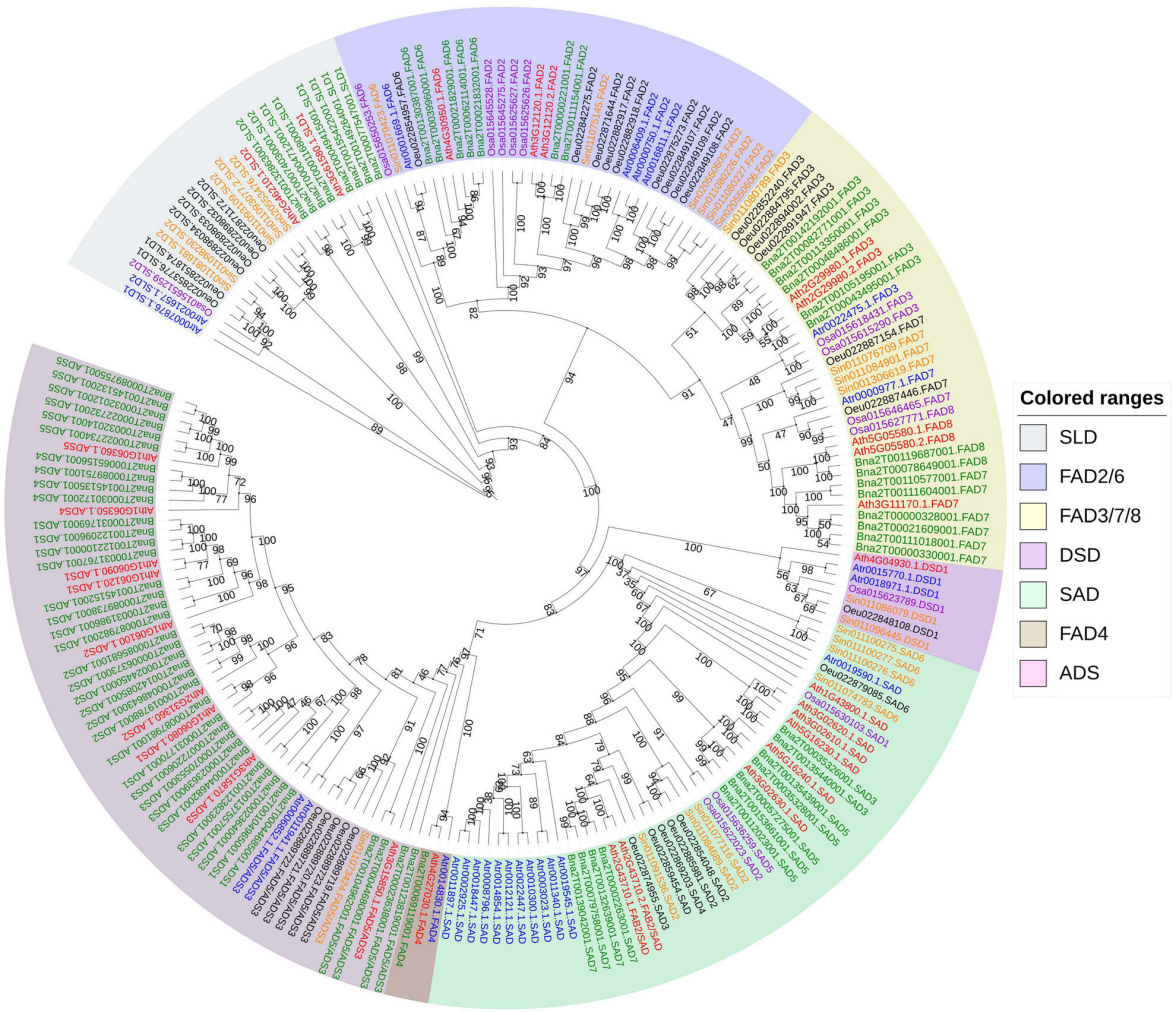
Phylogenetic relationship of FAD genes from *A. trifoliata* subsp. *australis*, *Arabidopsis thaliana*, *Oryza sativa*, *Sesamum indicum*, *Olea europaea*, and *Brassica rapa*. An ML phylogenetic tree of all detected FADs was constructed using RAxML with 1000 bootstrap replicates

A total of 25 FADs (acyl-lipid desaturase and acyl-ACP desaturase) in *A. trifoliata* were identified (Table S11). To evaluate their evolutionary relationships and predict their gene functions, all the *FAD* genes from *A. trifoliata*, *Arabidopsis*, rice, *Brassica napus*, *S. indicum*, and *O. europaea* were aligned to construct an unrooted ML phylogenetic tree (Fig. 8). All *AtrSADs*, except for *Atr0019590*, formed a well-defined monophyletic group, suggesting that *SAD* extension occurred after the divergence of *A. trifoliata* and other species. Notably, all identified *AtrFAD2* and *AtrFAD6* genes, as well as one *AtrSAD* (*Atr0019590*) gene, clustered with the homologs of *S. indicum* and *O. europaea*, which suggests that these genes perform similar functions in the three species. The expansion and neofunctionalization of *SADs* in oleasters are likely also responsible for the higher oleic acid contents in *A. trifoliata* than in sesame^17^.

In summary, genome sequencing of *A. trifoliata* has provided crucial information for the systematic study of the biosynthesis and metabolism of triterpenes in this medicinally and economically important nonmodel plant species. The *A. trifoliata* genome represents a useful resource for the genetic improvement of this plant species and for better understanding its genome evolution.

## Materials and methods

### Sampling and sequencing

Young *A. trifoliata* subsp. *australis* leaves were collected from Huaihua, Hunan Province, China (N27°33’17.95”, E109°59’54.70”). A modified cetyl trimethylammonium bromide method^44^ was used for DNA extraction. The DNA was used to construct a 20 kb insert-sized SMRTbell library for PacBio sequencing and 350 bp insert-size paired-end libraries for Illumina short-read sequencing. These libraries were prepared according to the manufacturer’s protocol (PacBio, CA, USA and Illumina, CA, USA). For RNA-seq, total RNA was extracted from the leaves, roots, and stems of *A. trifoliata*, and a mixture was made by combining an equal amount from each. After removing genomic DNA using DNase I (Takara), mRNAs were obtained using oligo (dT) beads and broken into short fragments, followed by cDNA synthesis. Paired-end sequencing was conducted on the HiSeq X Ten platform (Illumina, CA, USA). All PacBio and Illumina sequencing procedures were performed by the BioMarker Technologies Company (Beijing, China).

### Genome size estimation

Flow cytometry was used to estimate the genome size of *A. trifoliata* according to the method described by Huang et al.^45^. First, young fresh leaves of *A. trifoliata* were chopped in nuclear isolation buffer. The supernatant was filtered through a 50-μm CellTrics filter. After being treated with RNase, cell nuclei were stained with propidium iodide in the dark. The fluorescence intensity of the sample was determined using a flow cytometer (BD FACSVerse). Maize B73 was used as an internal standard. The genome size of *A. trifoliata* was evaluated by k-mer frequency analysis using Illumina short reads. After removing contaminants, the optimal k-mer size was analyzed using KmerGenie^46^. Then, Jellyfish was used to analyze the k-mer counts, which were used to estimate the genome size and heterozygosity^47^.

### Genome assembly and evaluation of genome quality

The PacBio Sequel long reads and Illumina short reads were combined to perform a de novo assembly of the *A. trifoliata* genome. Canu (v1.4) was first used to assemble the genome with the corrected-error-rate parameter^48^. Then, the corrected reads were independently assembled with WTDBG (v1.2.3)^49^. The well-assembled Canu and WTDBG results were merged by Quickmerge^50^. The merged genome was corrected with the Illumina data using Pilon^51^.

For the evaluation of assembly coverage, all paired-end short reads were mapped to our assembly using BWA^52^. RNA-Seq data from leaf, root, and stem tissues were assembled by Trinity^53^. HISAT2 was used to map all expressed sequence tags generated from RNA-Seq to the assembly with the default settings^54^ to evaluate gene completeness. The completeness of the assembly was assessed by the CEG mapping approach (v2.5) (CEGMA)^55^ and benchmarking universal single-copy ortholog (v4.0) (BUSCO) analysis^56^.

### Chromosome-scale assembly with Hi-C data

Hi-C libraries were constructed from DNA extracted from fresh leaf tissue of *A. trifoliata*, similar to what was used for genome assembly, as previously described^57^. The purified and enriched DNA was used for sequencing using the Illumina HiSeq X ten platform. A total of 103 Gb of clean data (151-fold the estimated genome size) was obtained and aligned to the PacBio assembly contigs using BOWTIE2^58^. The valid paired reads required for genome assembly were defined as uniquely mapped paired-end reads. Hi-C unique mapped paired-end reads were then applied to scaffold the assembled genome using the LACHESIS program^59^. The mapped read pairs were clustered into different chromosomal groups based on agglomerative hierarchical clustering. LACHESIS iterated all the possibilities of scaffold orientation and generated finely oriented scaffolds using a weighted directed acyclic graph.

### Genome annotation

Repeat elements were identified by combining de novo- and homology-based approaches. RepeatModeler^60^, LTR_Finder^61^, and RepeatScout^62^ were used to construct a repeat library for de novo prediction. Based on the repeat sequence database, homology prediction was conducted using RepeatProteinMask and RepeatMasker^63^ against the Repbase TE library^64^ and the TE protein database. Noncoding RNA was annotated using tRNAscan-SE^65^ (for tRNAs) or INFERNAL^66^ (for miRNAs and snRNAs). The rRNAs were identified using BLASTN alignment and RNAammer^67^.

Multiple gene prediction methods integrating homolog-, de novo-, and transcriptome-based gene prediction were used to annotate protein-coding genes. For homologous prediction, a gene set including proteins from four plant genomes (*Arabidopsis thaliana, A. coerulea, Opium poppy*, and *M. cordata*) was mapped to the assembly of the *A. trifoliata* genome by BLAST^68^, and then GeneWise software was used to provide an accurate gene structure prediction^69^. RNA-Seq reads were first aligned to our genome assembly using HISAT2^54^, and then StringTie^70^ was used to assemble the alignments into gene models in a transcriptome-based prediction. *De novo* identification was performed using Augustus (v2.5.5)^71^, GENSCAN (v1.0)^72^, SNAP^73^, and GlimmerHMM (v3.0.1)^74^. All the resulting genes mentioned above were integrated using EVidenceModeler^75^.

The protein-coding genes in *A. trifoliata* were blasted with an *E* value cutoff of 1.0 × 10^−5^ against SwissProt, NR, and the Kyoto Encyclopedia of Genes and Genomes (KEGG)^76^ for functional annotation. InterProScan (v4.8)^77^ and HMMER (v3.1)^78^ were used to annotate protein domains against the InterPro (v32.0)^79^ and Pfam (v27.0)^80^ databases, respectively. GO^81^ terms were obtained and grouped into three categories based on the results from the InterPro and Pfam entries.

### Dating of LTR retrotransposon elements

Intact LTR retrotransposons were identified by searching the genomes of *A. trifoliata* with LTR_Finder^61^ and LTR_STRUC^82^. According to the sequence divergence, the insertion times of the identified full-length LTR retrotransposons were estimated with Dismat (EMBOSS package)^83^. The average base substitution rate of 1.3E-08 per site per year was used to calculate the insertion times^84^.

### Gene family, phylogenetic analysis, and divergence time estimation

We collected the protein sequences from *A. trifoliata* and nine other plant species, namely, *A. thaliana*, *O. sativa*, *V. vinifera*, *A. coerulea*, *N. nucifera*, *P. somniferum*, *O. europaea*, *S. indicum*, and *A. trichopoda*, for gene family clustering. We conducted all-versus-all protein sequence queries through BLASTP with an *E* value of 1.0 × 10^−5^. OrthoMCL^85^ was used to cluster paralogous and orthologous groups. The four basal eudicot species, *A. trifoliata*, *A. coerulea*, *P. somniferum*, and *N. nucifera*, were further analyzed to explore their species-specific and shared gene families. The expansion and contraction of the gene family were analyzed using CAFE software^86^.

Following alignment by MUSCLE alignment software^87^, all single-copy genes identified and shared by the ten abovementioned species were used for evolutionary analysis and phylogenetic tree (ML Tree) reconstruction using RAxML software^88^. The divergence time was estimated using the MCMCtree program within the PAML package^89^. The divergence times were calibrated with the TimeTree database^90^.

### Whole-genome duplication analysis

Protein sequences were aligned against each other with BLASTP with an *E* value ≤ 1 × 10^−5^ to identify conserved paralogous and orthologous genes in *A. trifoliata, N. nucifera*, and *Vitis vinifera*. The 4DTv values were calculated using the HKY model^23^. Then, potential WGD events in the genome were evaluated based on the 4DTv value.

### Transcriptome analysis of developing seeds and qRT-PCR verification

*A. trifoliata* seeds were collected at 20, 50, 80, 110, and 140 days after flowering in May, June, July, August, and September, respectively. Library construction and RNA-Seq were performed as mentioned above. Clean reads were mapped to the reference genomes by HISAT2^54^. The expression level (fragments per kilobase of transcript per million fragments mapped, FPKM value) of unigenes was calculated by StringTie^65^. DEGs were identified by DESeq2^91^ (adjusted *P* value, FDR < 0.05). To clarify the biological functions of the DEGs, KEGG enrichment analysis was performed (http://www.genome.jp/kegg). Pathways with *P* < 0.05 were considered significantly enriched. A quantitative real-time polymerase chain reaction (qRT-PCR) assay was performed as described in Zahn et al.^22^. The *actin* gene was used as a reference in all experiments. Primers used for qRT-PCR are listed in Table S11. The qRT-PCR results were derived from three repeated reactions for each gene and sample. Fold change was calculated using the formula 2^−ΔΔCt^.

### Genome-wide identification and phylogenetic analysis of gene families

The genome and protein sequences of *A. thaliana, O. sativa, Vitis vinifera, O. europaea, S. indicum*, and *Brassica napus* were downloaded from the NCBI database. The hmmsearch program of HMMER software (version 3.2.1) (http://hmmer.org/download.html) was also applied to the identification of TPSs (PF01397, PF03936) and FADs (PF00487, PF03405) in Pfam 32.0 data (http://pfam.xfam.org/). To classify and investigate the phylogenetic relationships of the *amyrin synthase-like, TPS*, and *FAD* genes, the predicted genes were aligned using MUSCLE. Hence, two ML (maximum-likelihood) phylogenetic trees were constructed using RAxML software. The bootstrap test was performed with 1000 replicates to obtain high reliability of interior branches. The phylogenetic tree was imported to iTOL (https://itol.embl.de/) for visualization^92^.

### Assay of fatty-acid composition

The seeds at different developmental stages used in transcriptomic analysis were used to determine the FA composition. FA extraction and analysis were conducted according to Liu et al.^93^. Fifty milligrams of seed powder (fresh weight) was treated using chloroform-methanol solution (V/V = 2:1). Ultrasonic technology was used for FA extraction. Fatty acyl methyl esters (FAMEs) were prepared by direct transesterification of FA with 1% sulfuric acid in methanol at 80 °C for 30 min. The FAMEs were extracted with 1 mL hexane and analyzed by gas chromatography-mass spectrometry (GC-MS) with methyl heptadecanoate as an internal standard. GC-MS analysis was performed on an Agilent 6890 N/5975B (Agilent, USA) equipped with an Agilent HP-INNOWAX column (30 m × 0.25 mm ID × 0.25 µm). The column temperature was raised from 150 °C to 230 °C at a rate of 10 °C min^−1^ and then increased to 250 °C and maintained for 10 min. Peaks were identified by comparing the retention times with those of the corresponding standards (Sigma), and their identities were also confirmed by comparing mass spectra to the National Institute of Standards and Technology mass spectral library. The concentration of each sample was normalized according to the internal control.

Supplementary information accompanies the manuscript on the Horticulture Research website http://www.nature.com/hortres

## Supplementary information

Supplementary table S3 and S8

Supplementary figures and tables

## Data Availability

The *A. trifoliata* subsp. *australis* genome sequences, gene annoataion information, raw sequence data of genome sequencing and RNA-seq have been deposited under BioProject accession number PRJNA685604.
