# Supplementary material for: Insights into triterpene synthesis and unsaturated fatty-acid accumulation provided by chromosomal-level genome analysis of Akebia trifoliata subsp. australis
Source: Hortic Res. 2021 Feb 1;8:33. doi: 10.1038/s41438-020-00458-y (PMC7848005; doi:10.1038/s41438-020-00458-y)
Supplement: Supplementary file 2 — Supplementary figures and tables [file 41438_2020_458_MOESM2_ESM.doc]

**Supplementary Information Fig. S1. K-mer analysis for estimating the genome size of *Akebia trifoliata* subsp. *australis***. The paired-end reads from short insert-size libraries (350 bp) were used to generate the 19-mer frequency curve. The horizontal axis represents the K-mer depth, the number of times it occurred. The volume of K-mer was plotted against the frequency at which they occur. The left-head at low frequency and high volume represented K-mer containing essentially random sequencing errors. The main volume peak of K-mer was 31. Using the equation Genome size = (total number of k-mers)/(the volume peak), the genome size of *A. trifoliata* subsp. *australis* was estimated to be 20,951,554,382/31 = 669.76 Mb. The heterozygosity of the genome was 0.89%.


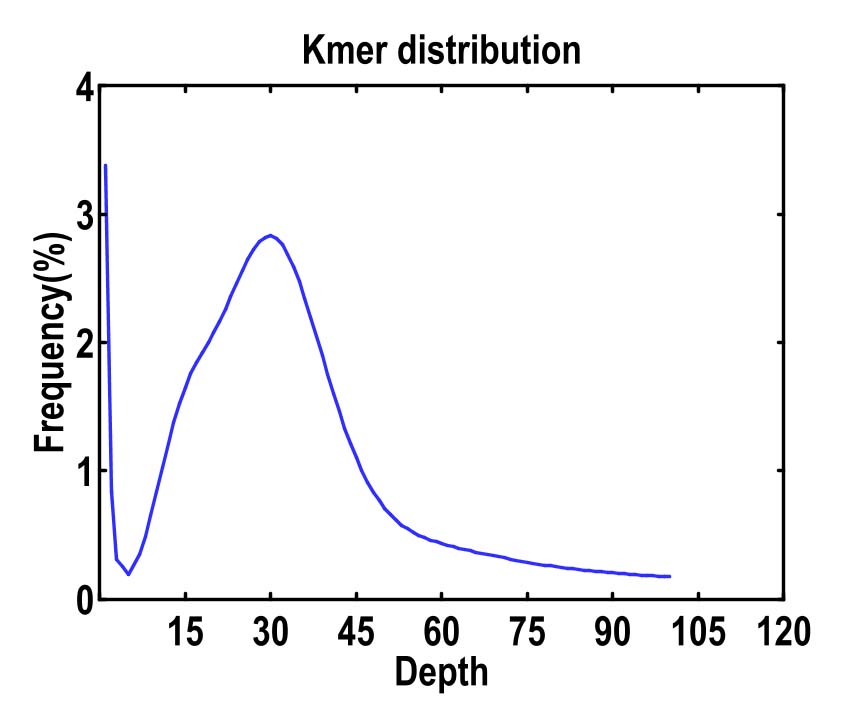


**Supplementary Information Fig. S2. Cytogram of fluorescence intensity of *Akebia trifoliata* subsp. *australis* and maize B73 nuclei.** Leaves of *A. trifoliata* subsp. *australis* and maize treated simultaneously processed (co-chopped) and stained with PI. **a** and **b**, two parameter histogram. **c**, single parameter histogram. X, Relative fluorescene. Y, Number of nuclei.


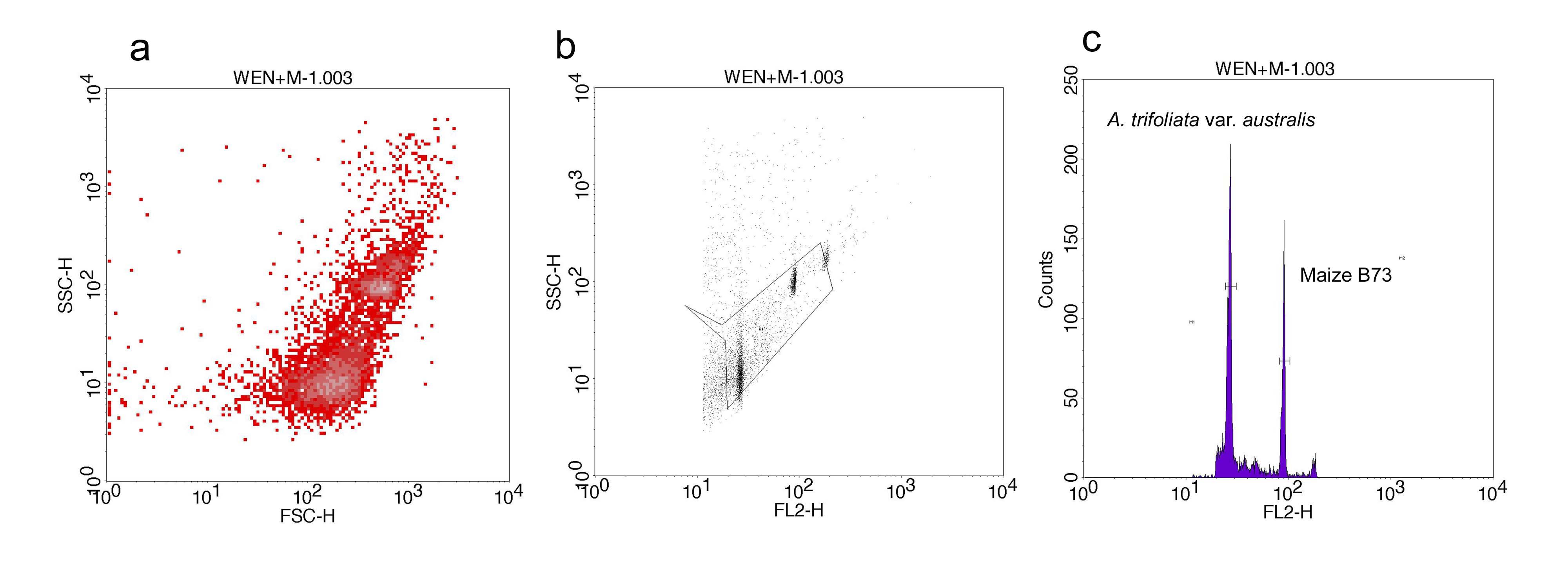


**Supplementary Information Fig. S3. Chromosome biology of *A. trifoliata* subsp. *australis*.** Basic fuchsin-stained young leaves showing the chromosome number (2n = 32). Three independent staining and counts were carried out.

**
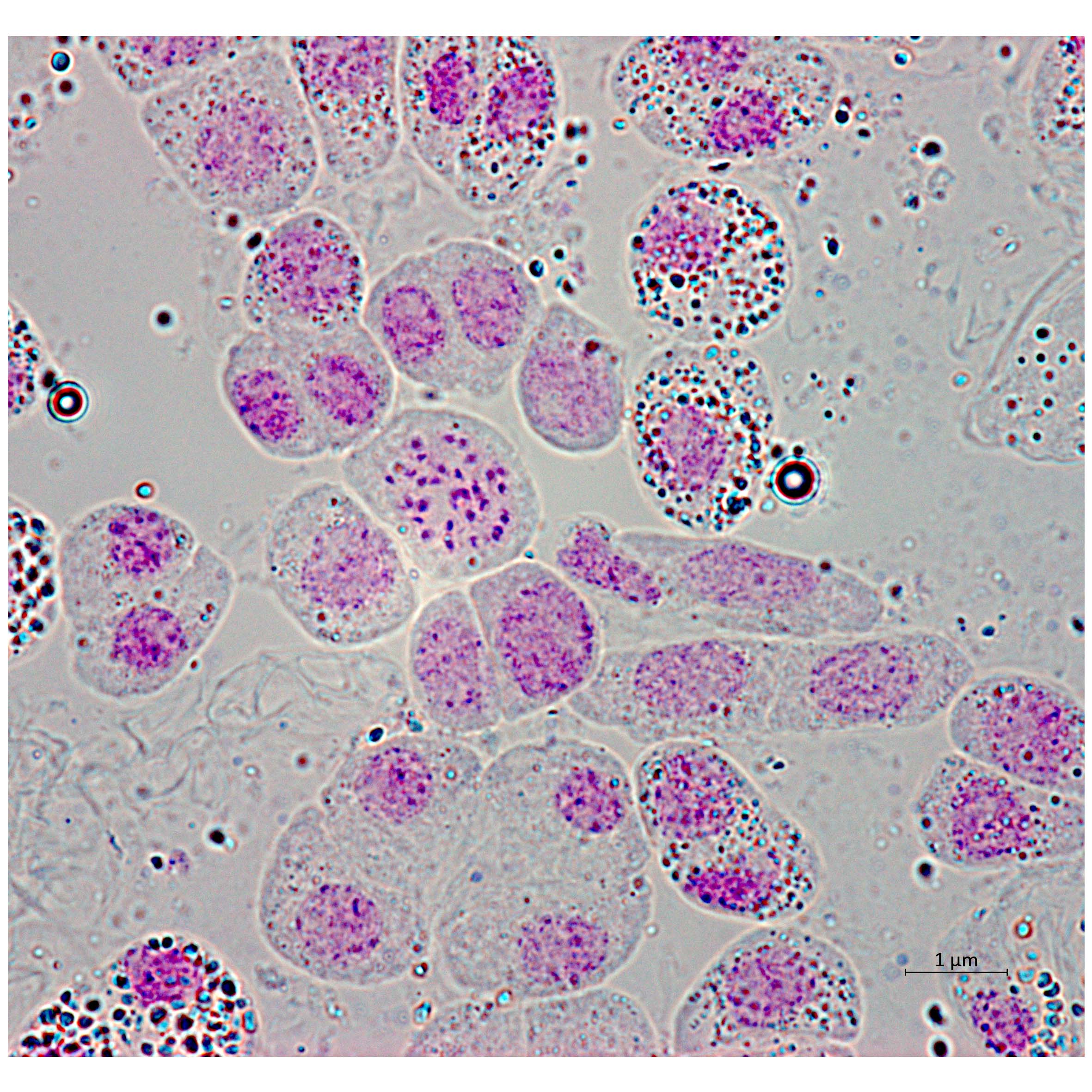
**

**Supplementary Information Fig. S4. The validation of completeness and base accuracy of the assembled *Akebia trifoliata* subsp. *australis* genome using (a) BUSCO and (b) CEGMA.**


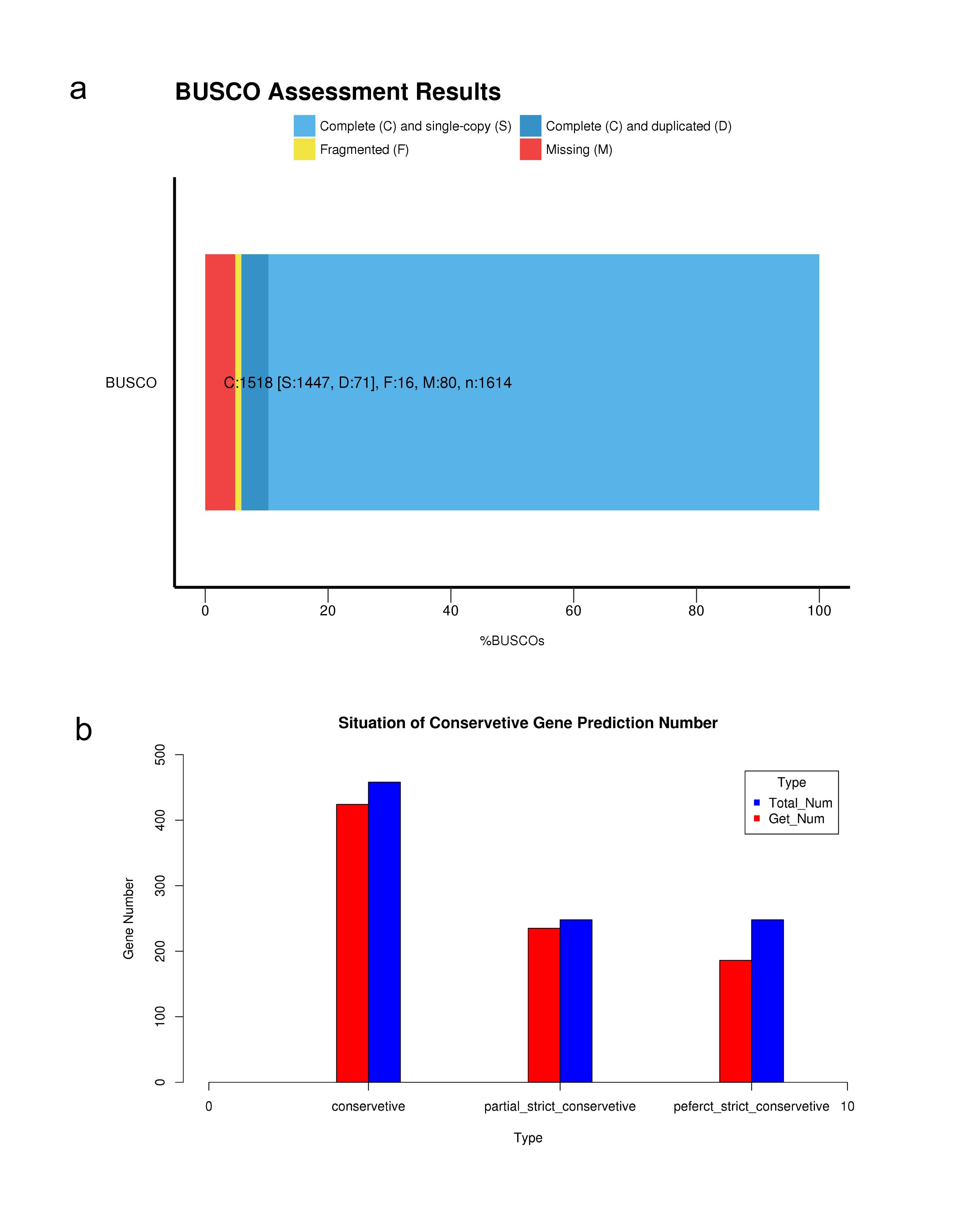


**Supplementary Information Fig. S5. Protein-coding gene annotation.** **a,** The number of protein-coding genes annotated by three methods. **b**, Nr homologous species distribution. **c**, KOG function classification. **d**, GO term of protein-coding genes.


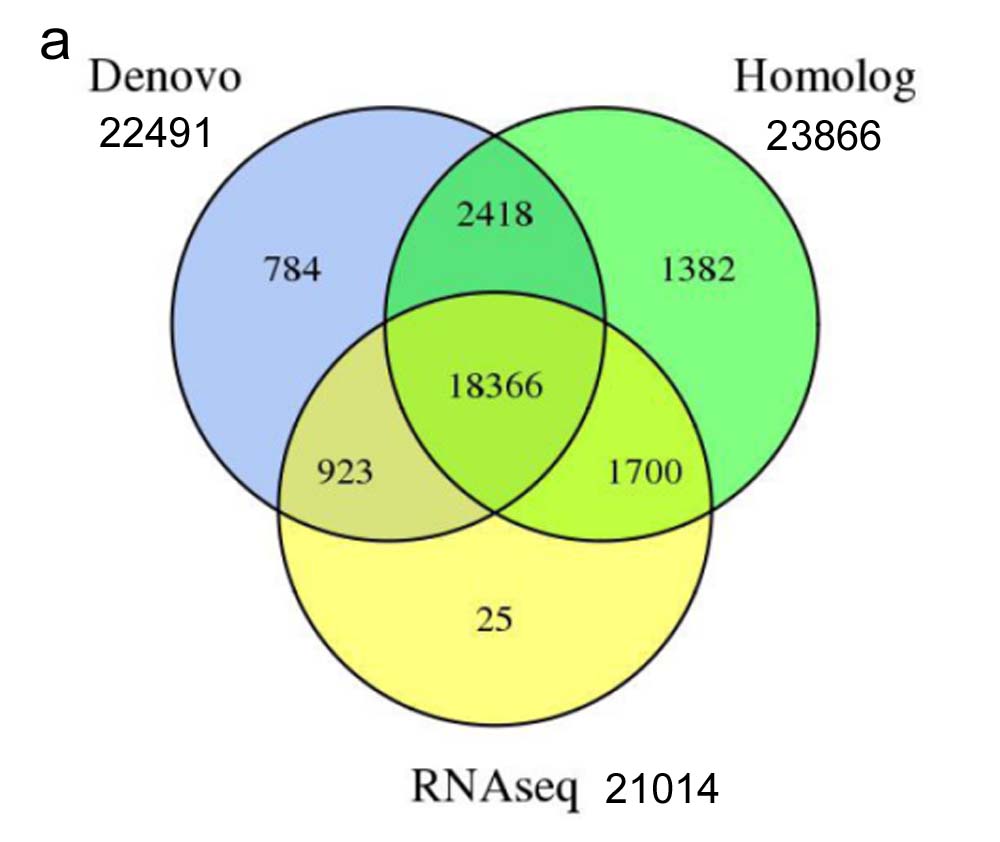


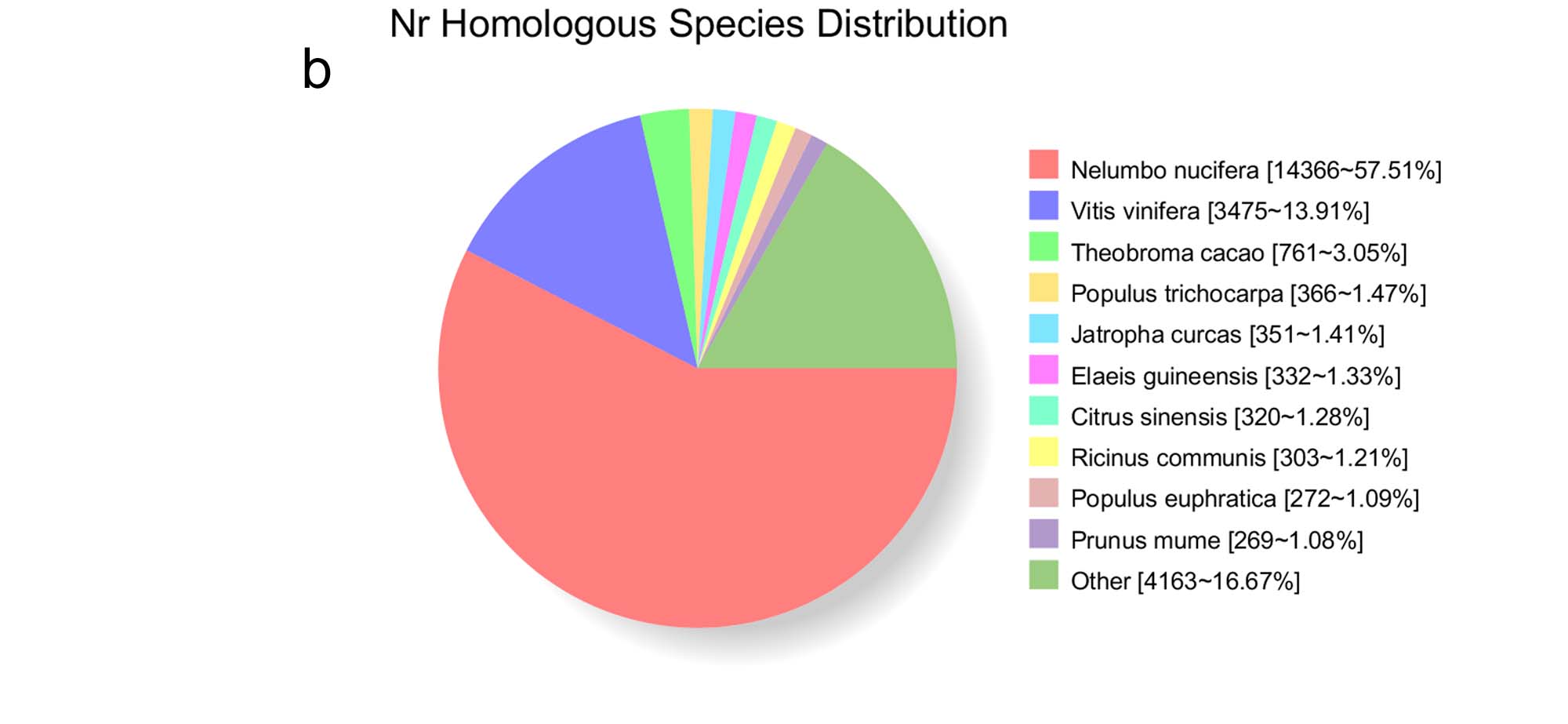


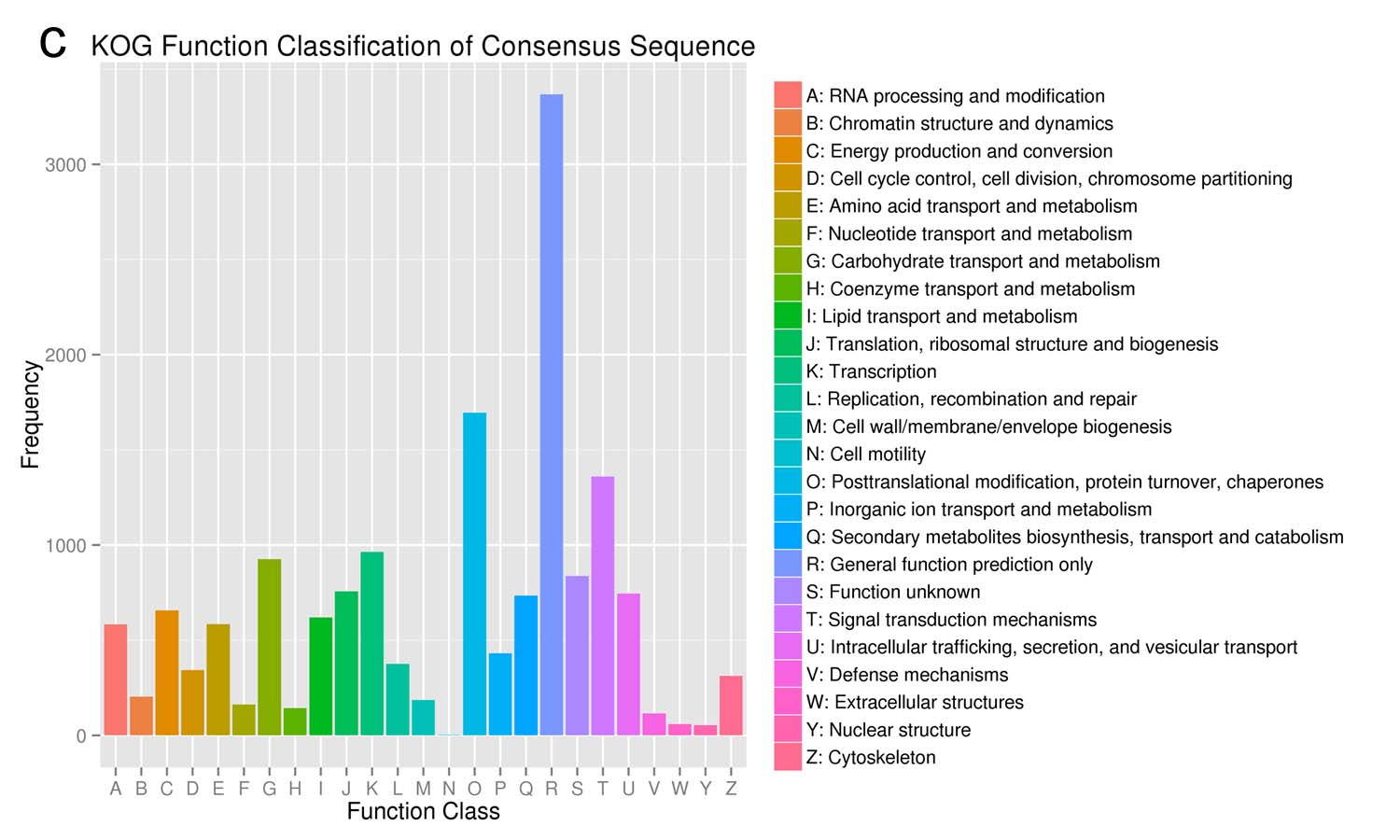


**
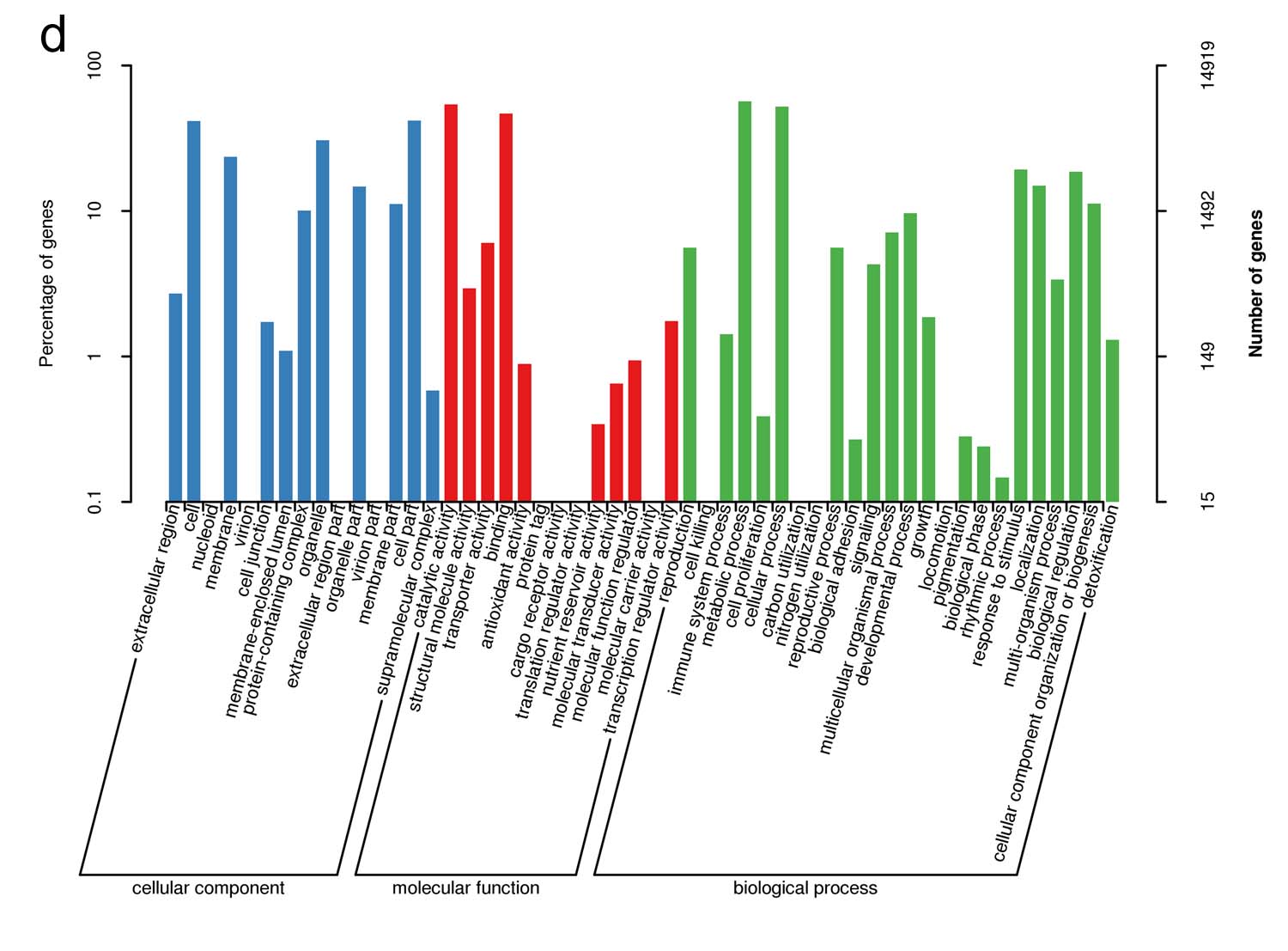
**

**Supplementary Information Fig. S6. GO analysis of the expanded (a) and contracted (b) genes**.

**
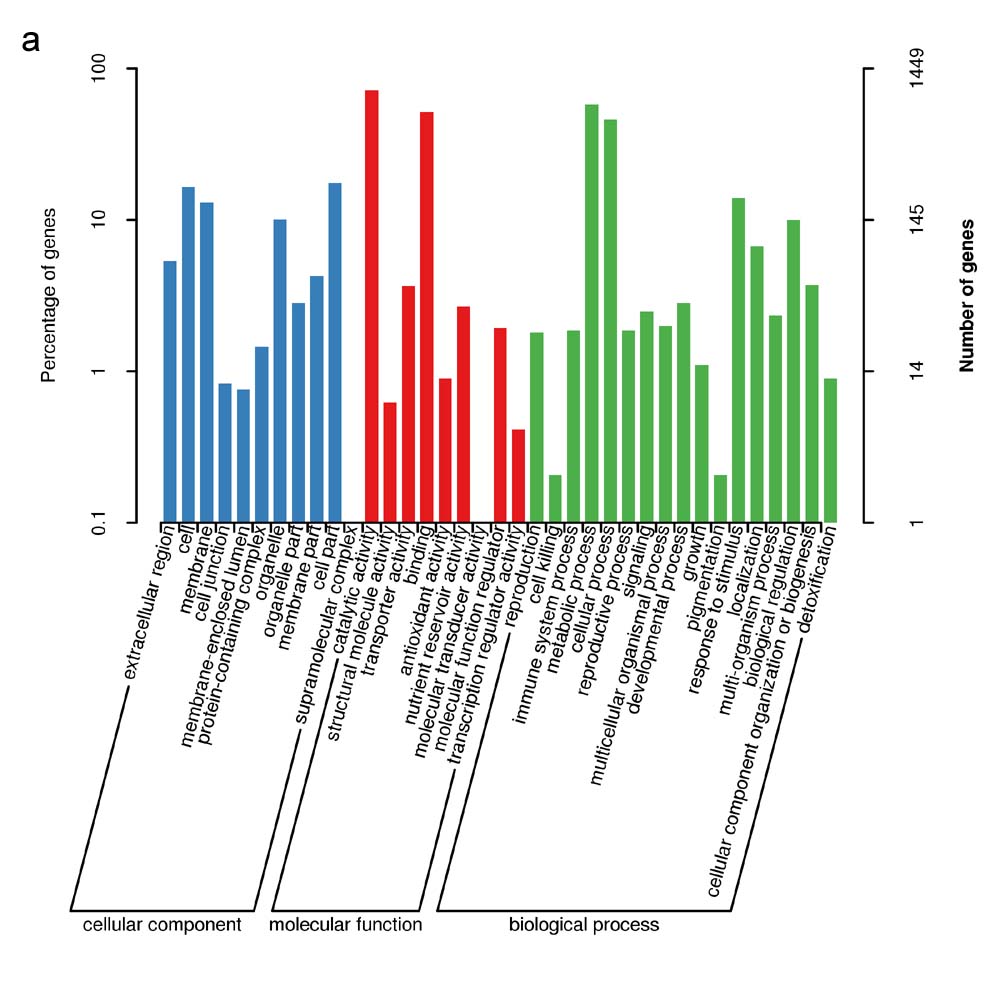
**

**
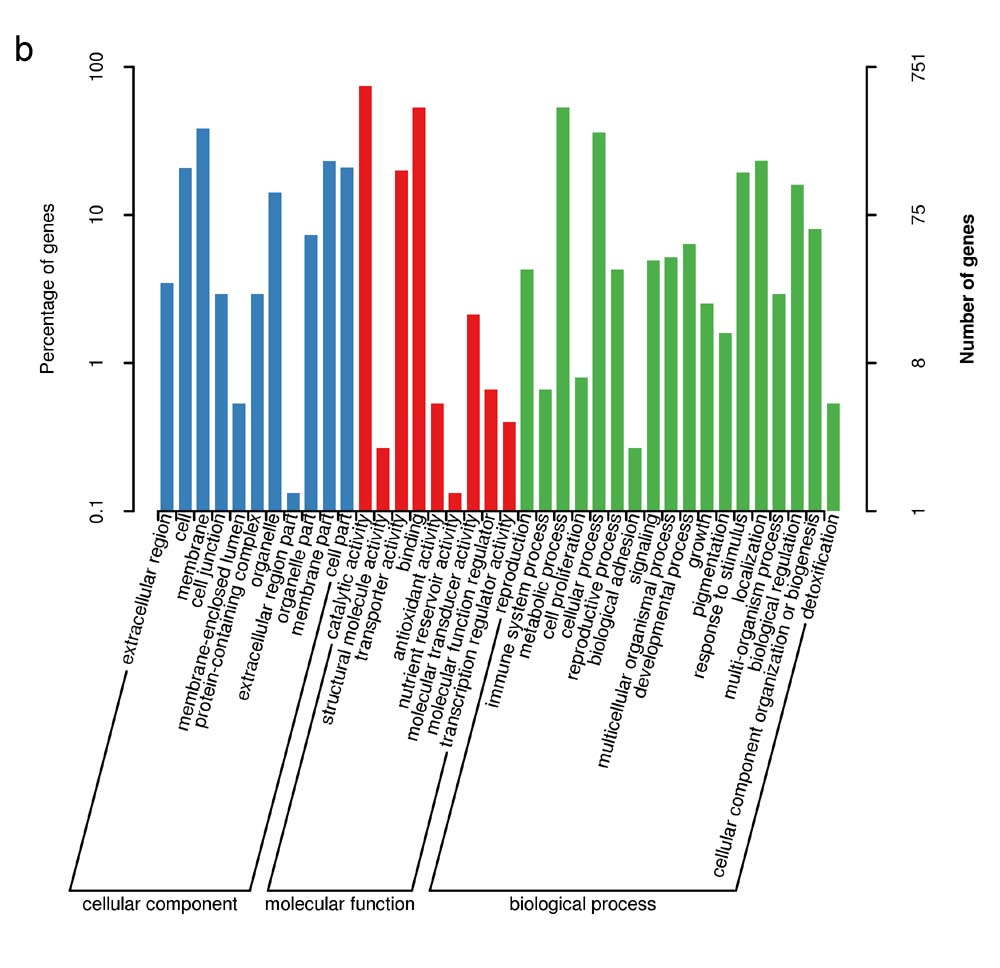
**

**Supplementary Information Fig. S7. phylogenetic tree of terpene synthase genes from *Akebia trifoliata* subsp. *australis, Arabidopsis,* rice and *V. vinifera*.** The phylogenetic tree was constructed using ML method with bootstrap of 1000 replicates.


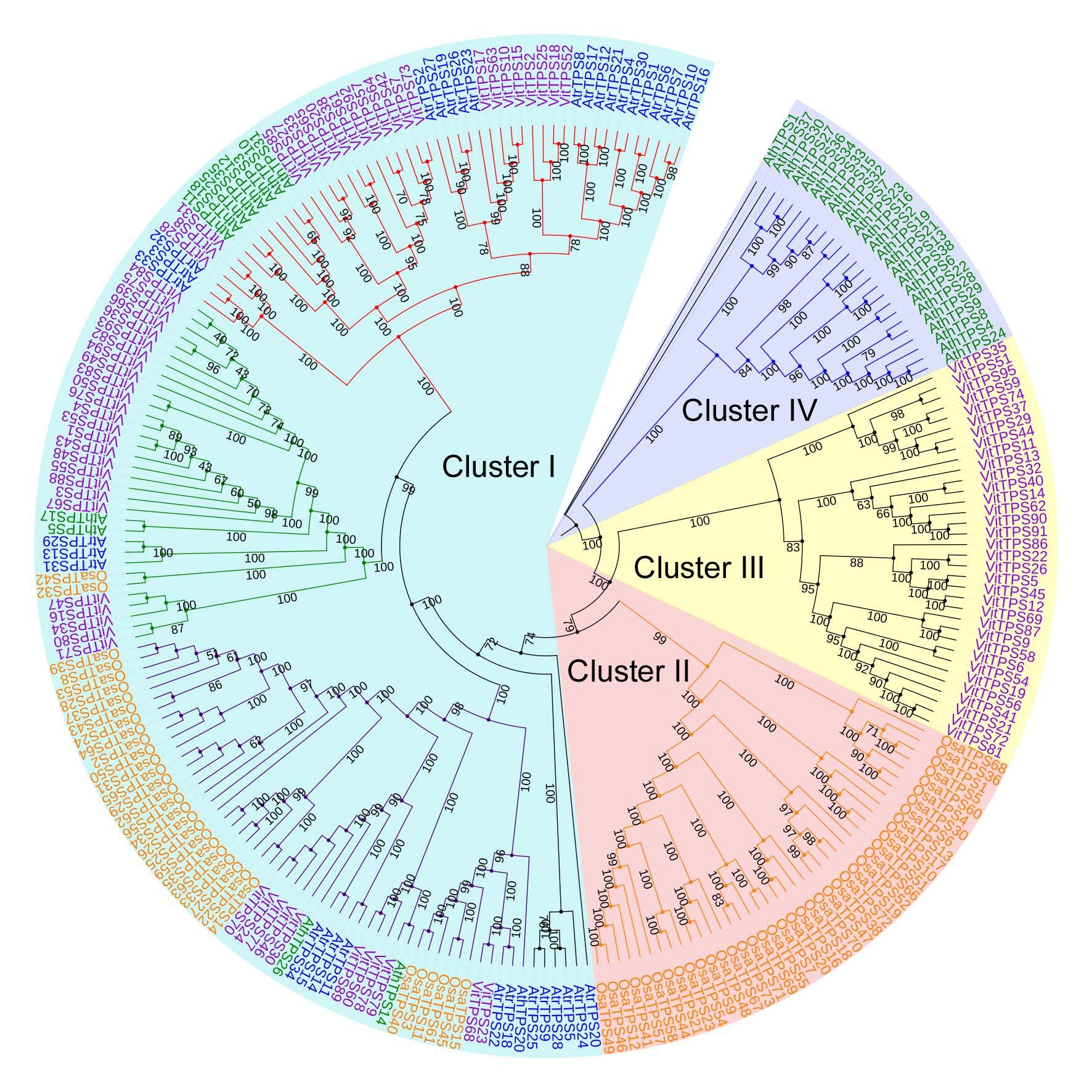


**Supplementary Information Fig. S8. KEGG annotations of the expanded (a) and contracted (b) genes.**

**
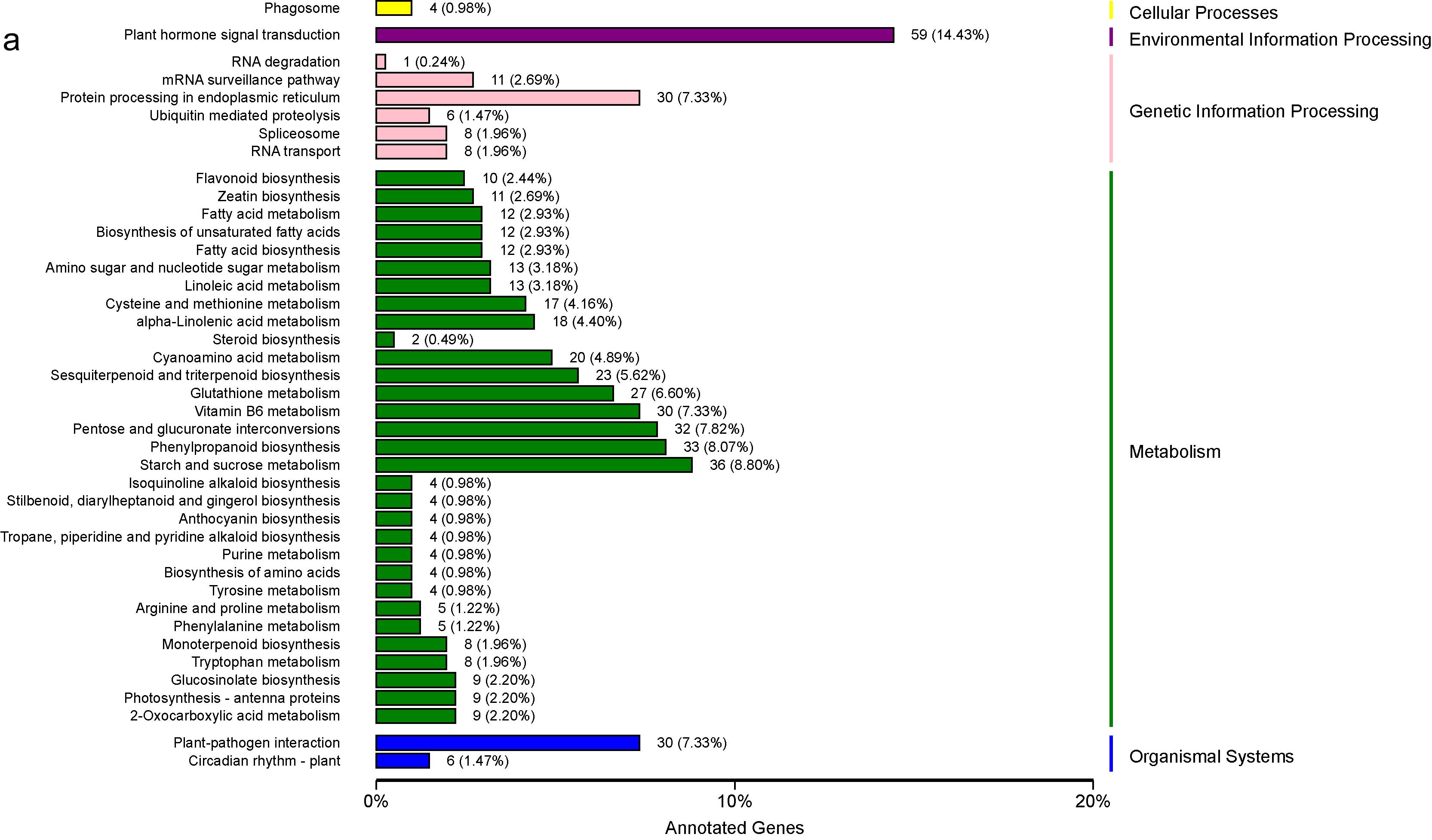
**

**
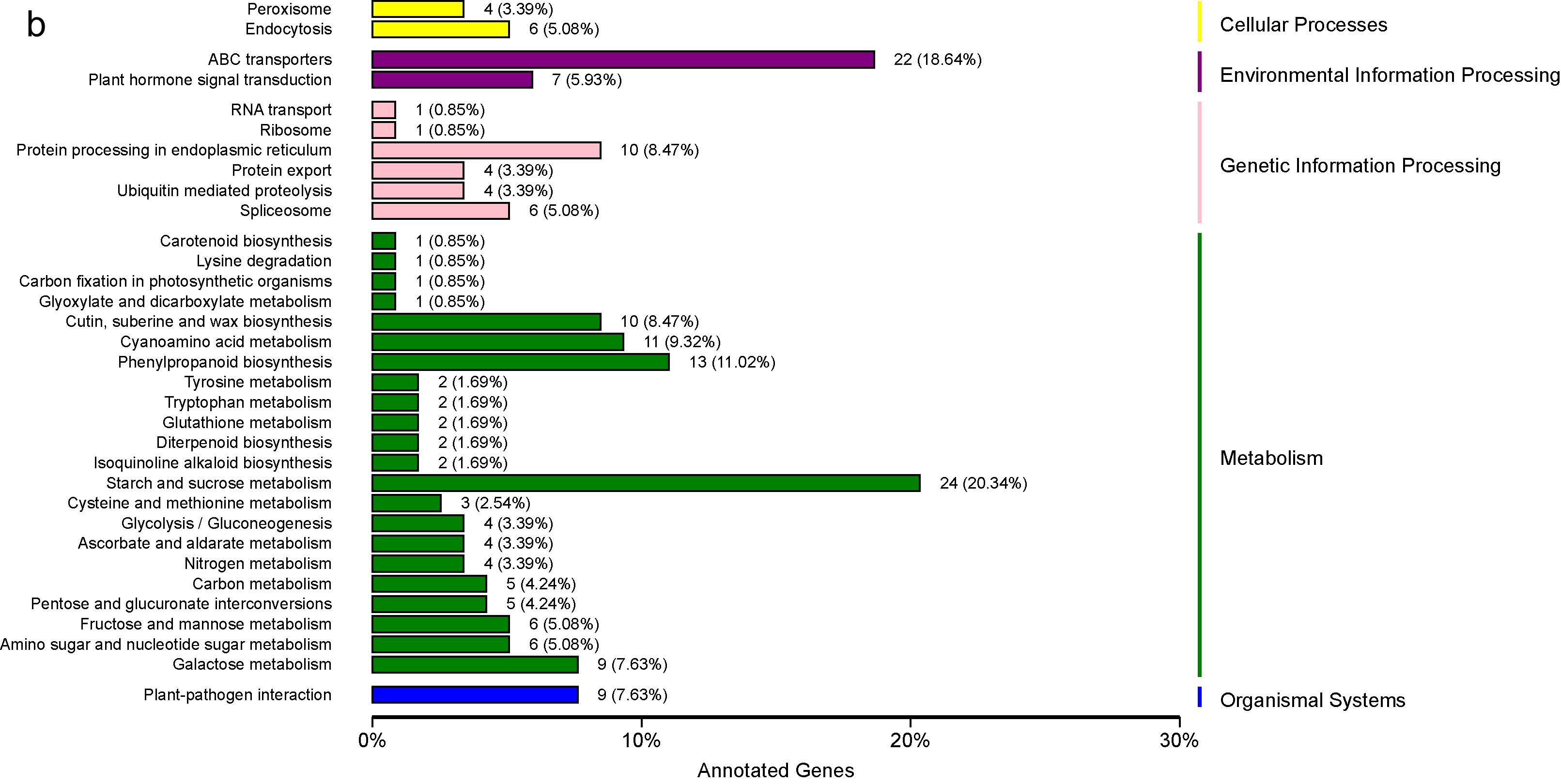
**

Supplementary Information Table S1. Sequencing data statistics and the assembly assessment results from CANU and WTDBG. The PacBio Sequel long-read sequencing with a 20-kb-insert-size SMRTbell library.

| **Cell name** | | | | | | **Productive ZMWs** | | | | **Productive ZMWs Productivity 0 (%)** | | | | | **Productivity 1 (%)** | | | | | **Productivity 2 (%)** | | |
| --- | --- | --- | --- | --- | --- | --- | --- | --- | --- | --- | --- | --- | --- | --- | --- | --- | --- | --- | --- | --- | --- | --- |
| m54155_190124_193209 | | | | | | 1016468 | | | | 27.79 | | | | | 62.51 | | | | | 9.71 | | |
| m54155_190128_165819 | | | | | | 1010188 | | | | 53.6 | | | | | 37.47 | | | | | 8.93 | | |
| m54237_190117_132933 | | | | | | 1012121 | | | | 61.75 | | | | | 31.11 | | | | | 7.14 | | |
| m54237_190118_162102 | | | | | | 1011367 | | | | 32.36 | | | | | 53.32 | | | | | 14.32 | | |
| m54237_190122_064117 | | | | | | 1009865 | | | | 17.8 | | | | | 66.36 | | | | | 15.83 | | |
| m54237_190124_094816 | | | | | | 1016821 | | | | 30.23 | | | | | 56.83 | | | | | 12.94 | | |
| m54237_190130_140402 | | | | | | 1018111 | | | | 53.9 | | | | | 40.18 | | | | | 5.92 | | |
| **Cell_name** | | | | | | **Reads_num** | | | **Total_base** | | | | **Read_N50** | | | | | **Mean** | | | **Maximum** | |
| m54155_190124_193209 | | | | | | 758378 | | | 10464974760 | | | | 22367 | | | | | 13799 | | | 82511 | |
| m54155_190128_165819 | | | | | | 475680 | | | 4649183011 | | | | 17356 | | | | | 9774 | | | 89133 | |
| m54237_190117_132933 | | | | | | 434308 | | | 6852345626 | | | | 25980 | | | | | 15778 | | | 90392 | |
| m54237_190118_162102 | | | | | | 668940 | | | 8739502231 | | | | 22566 | | | | | 13065 | | | 86860 | |
| m54237_190122_064117 | | | | | | 1056380 | | | 15759087833 | | | | 24304 | | | | | 14918 | | | 125076 | |
| m54237_190124_094816 | | | | | | 927387 | | | 14881092212 | | | | 25408 | | | | | 16046 | | | 121557 | |
| m54237_190130_140402 | | | | | | 449096 | | | 4647692551 | | | | 16949 | | | | | 10349 | | | 95709 | |
| **Length** | | | **Total_num_0** | | | | | | | | **Total_length_0** | | | | | | **Aver_length_0** | | | | | |
| 500~2000 | | | 485551 | | | | | | | | 626818616 | | | | | | 1290.94 | | | | | |
| 2000~4000 | | | 609432 | | | | | | | | 1797349817 | | | | | | 2949.22 | | | | | |
| 4000~6000 | | | 449114 | | | | | | | | 2225631873 | | | | | | 4955.61 | | | | | |
| 6000~8000 | | | 361543 | | | | | | | | 2519263597 | | | | | | 6968.09 | | | | | |
| 8000~10000 | | | 304072 | | | | | | | | 2728656562 | | | | | | 8973.72 | | | | | |
| 10000~12000 | | | 268026 | | | | | | | | 2944374614 | | | | | | 10985.41 | | | | | |
| 12000~14000 | | | 258498 | | | | | | | | 3360280236 | | | | | | 12999.25 | | | | | |
| 14000~16000 | | | 250608 | | | | | | | | 3755440001 | | | | | | 14985.32 | | | | | |
| 16000~18000 | | | 226165 | | | | | | | | 3840186820 | | | | | | 16979.58 | | | | | |
| 18000~ | | | 1418533 | | | | | | | | 42159445089 | | | | | | 29720.45 | | | | | |
| **Data_Type** | **Reads_num** | | | **Reads_base** | | | | **Reads_LenN50** | | | | | | **Reads_LenMean** | | | | | **Reads_LenMax** | | | |
| Subreads | 4631542 | | | 65957447225 | | | | 23030 | | | | | | 14241 | | | | | 125076 | | | |
| ZMWreads | 3408040 | | | 54356598254 | | | | 24877 | | | | | | 15950 | | | | | 125076 | | | |
| Contig number | | Contig length (bp) | | | Contig N50 (bp) | | Contig N90 (bp) | | | | | Contig max (bp) | | | | GC content (%) | | | | | | Gap total length (bp) |
| The assembly assessment results from CANU | | | | | | |  | | | | |  | | | |  | | | | | |  |
| 3468 | | 1153379919 | | | 818714 | | 115533 | | | | | 8822750 | | | | 34.95 | | | | | | 0 |
| The assembly assessment results from WTDBG | | | | | | |  | | | | |  | | | |  | | | | | |  |
| 1378 | | 637765077 | | | 2533610 | | 334441 | | | | | 13530042 | | | | 35.04 | | | | | | 0 |

**ZMW:** Zero-Mode Waveguides. **Productive ZMWs:** The number of ZMWs for this SMRT Cell that produced results with Productivity = 1. **Productivity 0 (%)**: Percentage of ZMWs that are empty, with no polymerase. **Productivity 1 (%):** Percentage of ZMWs that are productive and sequencing. **Productivity 2 (%):** Percentage of ZMWs that are not P0 (empty) or P1 (productive). This may occur for a variety of reasons and the sequence data is not usable.

**Supplementary Information Table S2.** General statistics of protein-coding genes annotation in *Akebia trifoliata* subsp. *australis* genome.

| **Anno_Database** | **Annotated_Number** | **Percentage (%)** |
| --- | --- | --- |
| GO_Annotation | 14919 | 58.28 |
| KEGG_Annotation | 9186 | 35.89 |
| KOG_Annotation | 14482 | 56.57 |
| Pfam_Annotation | 21015 | 82.1 |
| Swissprot_Annotation | 18498 | 72.26 |
| TrEMBL_Annotation | 24955 | 97.49 |
| nr_Annotation | 24979 | 97.58 |
| All_Annotated | 25008 | 97.7 |

Supplementary Information Table S4. Statistic of ncRNA in *A. trifoliata* var. *australis* genome.

| **rRNA_num** | **222** | **miRNA_num** | **97** |
| --- | --- | --- | --- |
| SSU_rRNA_eukarya | 26 | MIR171_1 | 17 |
| LSU_rRNA_eukarya | 31 | mir-395 | 3 |
| 5_8S_rRNA | 3 | mir-393 | 2 |
| 5S_rRNA | 162 | MIR396 | 2 |
| rRNA_cm_num | 222 | MIR398 | 1 |
| rRNA_fal_num | 4 | MIR169_2 | 9 |
|  |  | mir-172 | 8 |
| **tRNA_num** | **431** | mir-160 | 6 |
| Type=Tyr | 15 | MIR535 | 2 |
| Type=Pro | 34 | MIR394 | 1 |
| Type=Glu | 24 | MIR403 | 1 |
| Type=Met | 29 | MIR168 | 2 |
| Type=Thr | 21 | MIR408 | 2 |
| Type=Undet | 5 | MIR159 | 7 |
| Type=Asp | 23 | mir-166 | 7 |
| Type=Ser | 34 | MIR390 | 5 |
| Type=Gln | 19 | MIR171_2 | 2 |
| Type=Pseudo | 58 | mir-399 | 3 |
| Type=Trp | 12 | MIR473 | 2 |
| Type=Phe | 24 | mir-156 | 3 |
| Type=Cys | 13 | MIR164 | 3 |
| Type=Sup | 1 | MIR167_1 | 4 |
| Type=Gly | 37 | MIR169_5 | 5 |
| Type=Ala | 28 | miRNA_fal_num | 23 |
| Type=Arg | 33 |  |  |
| Type=His | 12 | **snRNA_num** | **94** |
| Type=Val | 23 | U1 | 25 |
| Type=Asn | 17 | U4 | 5 |
| Type=Ile | 24 | U6 | 26 |
| Type=Lys | 25 | U6atac | 16 |
| Type=Leu | 44 | U2 | 17 |
| tRNA_fal_num | 23 | U5 | 5 |
|  |  | snRNA_fal_num | 6 |
| **snoRNA_num** | **332** |  |  |
| HACA-box | 62 |  |  |
| CD-box | 270 |  |  |
| snoRNA_fal_num | 2 |  |  |

**Supplementary Information Table S5.** Summary of different types of transposable elements in the *A. trifoliata* subsp. *australis* genome.

| **Type** | **Number** | **Length (Mb)** | **Percent of assemble genome (%)** |
| --- | --- | --- | --- |
| ClassI/DIRS | 45445 | 23.5 | 3.45 |
| ClassI/LINE | 109193 | 31.8 | 4.67 |
| ClassI/LTR | 9315 | 4.5 | 0.66 |
| ClassI/LTR/Copia | 153797 | 64.8 | 9.5 |
| ClassI/LTR/Gypsy | 226617 | 220.9 | 32.38 |
| ClassI/PLE|LARD | 490609 | 141 | 20.66 |
| ClassI/SINE | 3646 | 0.8 | 0.11 |
| ClassI/TRIM | 5065 | 1.7 | 0.25 |
| ClassI/Unknown | 481 | 0.1 | 0.02 |
| ClassII/Crypton | 12 | 0.001 | 0 |
| ClassII/Helitron | 55545 | 15.1 | 2.21 |
| ClassII/MITE | 2045 | 0.3 | 0.04 |
| ClassII/Maverick | 12514 | 3.1 | 0.45 |
| ClassII/TIR | 64616 | 26.1 | 3.83 |
| ClassII/Unknown | 3198 | 0.5 | 0.08 |
| SSR | 634 | 0.1 | 0.02 |
| Unknown | 263344 | 67.3 | 9.86 |

**Supplementary Information Table S6.** Statistics of gene and gene families in *Akebia trifoliata* subsp. *australis* and other representative plant species.

| **Species name** | **Total gene number** | **oneCopy gene number** | **MultiCopy gene number** | **Unigene number** | **Other gene number** | **Cluster gene number** | **Uncluster gene number** | **Total family**  **number** | **Unique gene family number** |
| --- | --- | --- | --- | --- | --- | --- | --- | --- | --- |
| 1. *coerulea* | 30023 | 5149 | 6752 | 3430 | 8390 | 23721 | 6302 | 13468 | 983 |
| 1. ***trifoliata*** | **25598** | **4306** | **9074** | **1028** | **7997** | **22405** | **3193** | **12831** | **399** |
| 1. *somniferum* | 62879 | 495 | 22856 | 12089 | 23056 | 58496 | 4383 | 14513 | 2394 |
| 1. *nucifera* | 23940 | 3799 | 9361 | 798 | 7680 | 21638 | 2302 | 12483 | 259 |
| 1. *indicum* | 27148 | 4493 | 8124 | 3427 | 7013 | 23057 | 4091 | 12390 | 485 |
| *V. vinifera* | 26346 | 5208 | 6203 | 1876 | 5880 | 19167 | 7179 | 12188 | 652 |
| 1. *trichopoda* | 16986 | 5974 | 3624 | 759 | 4876 | 15233 | 1753 | 11444 | 232 |
| 1. *thaliana* | 27369 | 4457 | 8403 | 3140 | 7065 | 23065 | 4304 | 12418 | 847 |
| 1. *sativa* | 38852 | 4762 | 7797 | 7431 | 5572 | 25562 | 13290 | 12535 | 1930 |
| *O. europaea* | 39797 | 3175 | 13946 | 3290 | 12677 | 33088 | 6709 | 13463 | 815 |

**Supplementary Information Table S7. The expanded and contracted gene families in *Akebia trifoliata* subsp. *australis* genome compared with their most recent common ancestor (MRCA).**

| **GF_id** | **Pfam_id** | **Description** | **Expand (+) or Contracted (-)** |
| --- | --- | --- | --- |
| GF_151 | PF03087.9 | Arabidopsis protein of unknown function | - |
| GF_12822 | PF02365.10 | No apical meristem (NAM) protein | - |
| GF_511 | PF03492.10 | SAM dependent carboxyl methyltransferase | - |
| GF_43 | PF13456.1 | Reverse transcriptase-like | - |
| GF_331 | PF00043.20 | Glutathione S-transferase, C-terminal domain | - |
| GF_68 | PF13456.1 | Reverse transcriptase-like | - |
| GF_2257 | PF00646.28 | F-box domain | - |
| GF_180 | PF03006.15 | Haemolysin-III related | - |
| GF_113 | PF00078.22 | Reverse transcriptase (RNA-dependent DNA polymerase) | - |
| GF_2487 | PF00481.16 | Protein phosphatase 2C | - |
| GF_12818 | PF07730.8 | Histidine kinase | - |
| GF_348 | PF06839.7 | GRF zinc finger | - |
| GF_2259 | PF00319.13 | SRF-type transcription factor (DNA-binding and dimerisation domain) | - |
| GF_968 | PF04862.7 | Protein of unknown function (DUF642) | - |
| GF_181 | PF01095.14 | Pectinesterase | - |
| GF_75 | PF07734.8 | F-box associated | - |
| GF_121 | PF00931.17 | NB-ARC domain | - |
| GF_13391 | PF07893.8 | Protein of unknown function (DUF1668) | - |
| GF_2097 | PF00685.22 | Sulfotransferase domain | - |
| GF_7 | PF00931.17 | NB-ARC domain | - |
| GF_1216 | PF03106.10 | WRKY DNA -binding domain | - |
| GF_3862 | PF00891.13 | O-methyltransferase | - |
| GF_233 | PF00646.28 | F-box domain | - |
| GF_110 | PF08268.7 | F-box associated domain | - |
| GF_12066 | PF05773.17 | RWD domain | - |
| GF_1261 | PF01190.12 | Pollen proteins Ole e I like | - |
| GF_109 | PF14111.1 | Domain of unknown function (DUF4283) | - |
| GF_8614 | PF05938.6 | Plant self-incompatibility protein S1 | - |
| GF_45 | PF00854.16 | POT family | - |
| GF_8590 | PF00079.15 | Serpin (serine protease inhibitor) | - |
| GF_3267 | PF13639.1 | Ring finger domain | - |
| GF_503 | PF03016.10 | Exostosin family | - |
| GF_387 | PF12776.2 | Myb/SANT-like DNA-binding domain | - |
| GF_12277 | PF00646.28 | F-box domain | - |
| GF_27 | PF00078.22 | Reverse transcriptase (RNA-dependent DNA polymerase) | - |
| GF_2395 | PF03007.11 | Wax ester synthase-like Acyl-CoA acyltransferase domain | - |
| GF_8585 | PF07734.8 | F-box associated | - |
| GF_259 | PF13837.1 | Myb/SANT-like DNA-binding domain | - |
| GF_12836 | PF00069.20 | Protein kinase domain | - |
| GF_12624 | PF13083.1 | KH domain | - |
| GF_239 | PF07734.8 | F-box associated | - |
| GF_38 | PF00067.17 | Cytochrome P450 | - |
| GF_11609 | PF13639.1 | Ring finger domain | - |
| GF_232 | PF00232.13 | Glycosyl hydrolase family 1 | - |
| GF_1805 | PF10354.4 | Domain of unknown function (DUF2431) | - |
| GF_25 | PF10551.4 | MULE transposase domain | - |
| GF_59 | PF00078.22 | Reverse transcriptase (RNA-dependent DNA polymerase) | - |
| GF_3193 | PF10267.4 | Predicted transmembrane and coiled-coil 2 protein | - |
| GF_135 | PF03094.10 | Mlo family | - |
| GF_844 | PF02485.16 | Core-2/I-Branching enzyme | - |
| GF_429 | PF00646.28 | F-box domain | - |
| GF_11592 | PF00072.19 | Response regulator receiver domain | - |
| GF_163 | PF02373.17 | JmjC domain, hydroxylase | - |
| GF_481 | PF07859.8 | alpha/beta hydrolase fold | - |
| GF_12833 | PF13041.1 | PPR repeat family | - |
| GF_11825 | PF00891.13 | O-methyltransferase | - |
| GF_298 | PF00332.13 | Glycosyl hydrolases family 17 | - |
| GF_1548 | PF03151.11 | Triose-phosphate Transporter family | - |
| GF_12282 | PF00646.28 | F-box domain | - |
| GF_1802 | PF00314.12 | Thaumatin family | - |
| GF_157 | PF07727.9 | Reverse transcriptase (RNA-dependent DNA polymerase) | - |
| GF_282 | PF07714.12 | Protein tyrosine kinase | - |
| GF_365 | PF00443.24 | Ubiquitin carboxyl-terminal hydrolase | - |
| GF_510 | PF08214.6 | Histone acetylation protein | - |
| GF_10386 | PF08268.7 | F-box associated domain | - |
| GF_12824 | PF00083.19 | Sugar (and other) transporter | - |
| GF_212 | PF00067.17 | Cytochrome P450 | - |
| GF_1402 | PF03478.13 | Protein of unknown function (DUF295) | - |
| GF_12622 | PF13437.1 | HlyD family secretion protein | - |
| GF_140 | PF14291.1 | Domain of unknown function (DUF4371) | - |
| GF_13411 | PF07734.8 | F-box associated | - |
| GF_123 | PF05691.7 | Raffinose synthase or seed imbibition protein Sip1 | - |
| GF_12015 | PF00071.17 | Ras family | - |
| GF_608 | PF00319.13 | SRF-type transcription factor (DNA-binding and dimerisation domain) | - |
| GF_11588 | PF13837.1 | Myb/SANT-like DNA-binding domain | - |
| GF_2381 | PF04059.7 | RNA recognition motif 2 | - |
| GF_3767 | PF05266.9 | Protein of unknown function (DUF724) | - |
| GF_315 | PF00125.19 | Core histone H2A/H2B/H3/H4 | - |
| GF_20 | PF04827.9 | Plant transposon protein | - |
| GF_51 | PF00069.20 | Protein kinase domain | - |
| GF_12813 | PF14299.1 | Phloem protein 2 | - |
| GF_55 | PF07731.9 | Multicopper oxidase | - |
| GF_13383 | PF13855.1 | Leucine rich repeat | - |
| GF_2801 | PF05922.11 | Peptidase inhibitor I9 | - |
| GF_13368 | PF05266.9 | Protein of unknown function (DUF724) | - |
| GF_2514 | PF08646.5 | Replication factor-A C terminal domain | - |
| GF_654 | PF00400.27 | WD domain, G-beta repeat | - |
| GF_11638 | PF12224.3 | Putative amidoligase enzyme | - |
| GF_8528 | PF00069.20 | Protein kinase domain | - |
| GF_186 | PF02458.10 | Transferase family | - |
| GF_748 | PF00295.12 | Glycosyl hydrolases family 28 | - |
| GF_12301 | PF00011.16 | Hsp20/alpha crystallin family | - |
| GF_5483 | PF14226.1 | non-haem dioxygenase in morphine synthesis N-terminal | - |
| GF_11590 | PF00646.28 | F-box domain | - |
| GF_2272 | PF00264.15 | Common central domain of tyrosinase | - |
| GF_861 | PF03140.10 | Plant protein of unknown function | - |
| GF_3279 | PF00931.17 | NB-ARC domain | - |
| GF_1155 | PF02182.12 | SAD/SRA domain | - |
| GF_10503 | PF03004.9 | Plant transposase (Ptta/En/Spm family) | - |
| GF_13798 | PF08387.5 | FBD | - |
| GF_767 | PF12697.2 | Alpha/beta hydrolase family | - |
| GF_37 | PF00078.22 | Reverse transcriptase (RNA-dependent DNA polymerase) | - |
| GF_201 | PF00234.17 | Protease inhibitor/seed storage/LTP family | - |
| GF_606 | PF07734.8 | F-box associated | - |
| GF_2312 | PF02892.10 | BED zinc finger | - |
| GF_13152 | PF13447.1 | Seven times multi-haem cytochrome CxxCH | - |
| GF_1283 | PF02362.16 | B3 DNA binding domain | - |
| GF_1053 | PF08646.5 | Replication factor-A C terminal domain | - |
| GF_15 | PF00931.17 | NB-ARC domain | - |
| GF_206 | PF03727.11 | Hexokinase | - |
| GF_4384 | PF02362.16 | B3 DNA binding domain | - |
| GF_21 | PF13041.1 | PPR repeat family | - |
| GF_780 | PF03478.13 | Protein of unknown function (DUF295) | - |
| GF_13376 | PF00120.19 | Glutamine synthetase, catalytic domain | - |
| GF_220 | PF00190.17 | Cupin | - |
| GF_11596 | PF02365.10 | No apical meristem (NAM) protein | - |
| GF_142 | PF01061.19 | ABC-2 type transporter | - |
| GF_248 | PF13962.1 | Domain of unknown function | - |
| GF_60 | PF10536.4 | Plant mobile domain | - |
| GF_6175 | PF05617.6 | Prolamin-like | - |
| GF_4377 | PF02902.14 | Ulp1 protease family, C-terminal catalytic domain | - |
| GF_402 | PF03514.9 | GRAS domain family | - |
| GF_8603 | PF03080.10 | Domain of unknown function (DUF239) | - |
| GF_901 | PF06830.6 | Root cap | - |
| GF_714 | PF00069.20 | Protein kinase domain | - |
| GF_854 | PF02458.10 | Transferase family | - |
| GF_12570 | PF00730.20 | HhH-GPD superfamily base excision DNA repair protein | - |
| GF_108 | PF01554.13 | MatE | - |
| GF_3376 | PF00249.26 | Myb-like DNA-binding domain | - |
| GF_11574 | PF07734.8 | F-box associated | - |
| GF_1526 | PF08387.5 | FBD | - |
| GF_1142 | PF01485.16 | IBR domain | - |
| GF_12831 | PF00657.17 | GDSL-like Lipase/Acylhydrolase | - |
| GF_361 | PF10551.4 | MULE transposase domain | - |
| GF_12845 | PF00304.15 | Gamma-thionin family | - |
| GF_471 | PF13456.1 | Reverse transcriptase-like | - |
| GF_533 | PF01466.14 | Skp1 family, dimerisation domain | - |
| GF_205 | PF10551.4 | MULE transposase domain | - |
| GF_937 | PF07649.7 | C1-like domain | - |
| GF_575 | PF03106.10 | WRKY DNA -binding domain | - |
| GF_111 | PF00407.14 | Pathogenesis-related protein Bet v I family | - |
| GF_1183 | PF07734.8 | F-box associated | - |
| GF_13366 | PF07876.7 | Stress responsive A/B Barrel Domain | - |
| GF_19 | PF10551.4 | MULE transposase domain | - |
| GF_6170 | PF03469.9 | XH domain | - |
| GF_3261 | PF12796.2 | Ankyrin repeats (3 copies) | - |
| GF_623 | PF03151.11 | Triose-phosphate Transporter family | - |
| GF_11929 | PF04043.10 | Plant invertase/pectin methylesterase inhibitor | - |
| GF_502 | PF01485.16 | IBR domain | - |
| GF_1054 | PF11443.3 | Domain of unknown function (DUF2828) | - |
| GF_2802 | PF00646.28 | F-box domain | - |
| GF_1571 | PF13964.1 | Kelch motif | - |
| GF_4974 | PF00407.14 | Pathogenesis-related protein Bet v I family | - |
| GF_2826 | PF03140.10 | Plant protein of unknown function | - |
| GF_427 | PF00145.12 | C-5 cytosine-specific DNA methylase | - |
| GF_2511 | PF03650.8 | Uncharacterised protein family (UPF0041) | - |
| GF_12815 | PF00646.28 | F-box domain | - |
| GF_856 | PF03330.13 | Rare lipoprotein A (RlpA)-like double-psi beta-barrel | - |
| GF_189 | PF00069.20 | Protein kinase domain | - |
| GF_725 | PF00407.14 | Pathogenesis-related protein Bet v I family | - |
| GF_18 | PF07500.9 | Transcription factor S-II (TFIIS), central domain | - |
| GF_12300 | PF00319.13 | SRF-type transcription factor (DNA-binding and dimerisation domain) | - |
| GF_300 | PF12697.2 | Alpha/beta hydrolase family | - |
| GF_353 | PF00854.16 | POT family | - |
| GF_47 | PF14244.1 | gag-polypeptide of LTR copia-type | - |
| GF_369 | PF12076.3 | WAX2 C-terminal domain | - |
| GF_215 | PF00931.17 | NB-ARC domain | - |
| GF_3777 | PF02469.17 | Fasciclin domain | - |
| GF_10421 | PF12937.2 | F-box-like | - |
| GF_458 | PF05686.7 | Glycosyl transferase family 90 | - |
| GF_7288 | PF14111.1 | Domain of unknown function (DUF4283) | - |
| GF_446 | PF08268.7 | F-box associated domain | - |
| GF_32 | PF00664.18 | ABC transporter transmembrane region | - |
| GF_332 | PF00685.22 | Sulfotransferase domain | - |
| GF_617 | PF03171.15 | 2OG-Fe(II) oxygenase superfamily | - |
| GF_13409 | PF07734.8 | F-box associated | - |
| GF_4394 | PF14111.1 | Domain of unknown function (DUF4283) | - |
| GF_6195 | PF02362.16 | B3 DNA binding domain | - |
| GF_9115 | PF05970.9 | PIF1-like helicase | - |
| GF_13794 | PF01497.13 | Periplasmic binding protein | - |
| GF_508 | PF00646.28 | F-box domain | - |
| GF_6973 | PF05970.9 | PIF1-like helicase | - |
| GF_13390 | PF06161.6 | Protein of unknown function (DUF975) | - |
| GF_30 | PF10551.4 | MULE transposase domain | - |
| GF_92 | PF13855.1 | Leucine rich repeat | - |
| GF_857 | PF12819.2 | Carbohydrate-binding protein of the ER | - |
| GF_1625 | PF00067.17 | Cytochrome P450 | - |
| GF_274 | PF13360.1 | PQQ-like domain | - |
| GF_10354 | PF00319.13 | SRF-type transcription factor (DNA-binding and dimerisation domain) | - |
| GF_6227 | PF14497.1 | Glutathione S-transferase, C-terminal domain | - |
| GF_614 | PF00657.17 | GDSL-like Lipase/Acylhydrolase | - |
| GF_386 | PF00892.15 | EamA-like transporter family | - |
| GF_2047 | PF08268.7 | F-box associated domain | - |
| GF_179 | PF00759.14 | Glycosyl hydrolase family 9 | - |
| GF_160 | PF00650.15 | CRAL/TRIO domain | - |
| GF_12850 | PF14543.1 | Xylanase inhibitor N-terminal | - |
| GF_83 | PF01490.13 | Transmembrane amino acid transporter protein | - |
| GF_13396 | PF03004.9 | Plant transposase (Ptta/En/Spm family) | - |
| GF_376 | PF14303.1 | No apical meristem-associated C-terminal domain | - |
| GF_10437 | PF04504.9 | Protein of unknown function, DUF573 | - |
| GF_221 | PF10551.4 | MULE transposase domain | - |
| GF_351 | PF13519.1 | von Willebrand factor type A domain | - |
| GF_12276 | PF12937.2 | F-box-like | - |
| GF_1523 | PF08646.5 | Replication factor-A C terminal domain | - |
| GF_4408 | PF13912.1 | C2H2-type zinc finger | - |
| GF_187 | PF02458.10 | Transferase family | - |
| GF_500 | PF01554.13 | MatE | - |
| GF_676 | PF03254.8 | Xyloglucan fucosyltransferase | - |
| GF_5258 | PF02353.15 | Mycolic acid cyclopropane synthetase | - |
| GF_96 | PF00665.21 | Integrase core domain | - |
| GF_11589 | PF07734.8 | F-box associated | - |
| GF_3234 | PF02601.10 | Exonuclease VII, large subunit | - |
| GF_1528 | PF03007.11 | Wax ester synthase-like Acyl-CoA acyltransferase domain | - |
| GF_1785 | PF01055.21 | Glycosyl hydrolases family 31 | - |
| GF_3235 | PF00067.17 | Cytochrome P450 | - |
| GF_2520 | PF14144.1 | Seed dormancy control | - |
| GF_12267 | PF01453.19 | D-mannose binding lectin | - |
| GF_12268 | PF12776.2 | Myb/SANT-like DNA-binding domain | - |
| GF_948 | PF00891.13 | O-methyltransferase | - |
| GF_11620 | PF08268.7 | F-box associated domain | - |
| GF_34 | PF01061.19 | ABC-2 type transporter | - |
| GF_7299 | PF00240.18 | Ubiquitin family | - |
| GF_2998 | PF08268.7 | F-box associated domain | - |
| GF_93 | PF00067.17 | Cytochrome P450 | - |
| GF_97 | PF00067.17 | Cytochrome P450 | - |
| GF_556 | PF00067.17 | Cytochrome P450 | - |
| GF_12458 | PF00722.16 | Glycosyl hydrolases family 16 | - |
| GF_2791 | PF00249.26 | Myb-like DNA-binding domain | - |
| GF_886 | PF00043.20 | Glutathione S-transferase, C-terminal domain | - |
| GF_515 | PF07690.11 | Major Facilitator Superfamily | - |
| GF_1009 | PF03184.14 | DDE superfamily endonuclease | - |
| GF_22 | PF00664.18 | ABC transporter transmembrane region | - |
| GF_13795 | PF13520.1 | Amino acid permease | - |
| GF_2222 | PF13962.1 | Domain of unknown function | - |
| GF_437 | PF02362.16 | B3 DNA binding domain | - |
| GF_677 | PF13855.1 | Leucine rich repeat | - |
| GF_162 | PF14223.1 | gag-polypeptide of LTR copia-type | - |
| GF_33 | PF00083.19 | Sugar (and other) transporter | - |
| GF_1506 | PF00847.15 | AP2 domain | - |
| GF_10442 | PF00650.15 | CRAL/TRIO domain | - |
| GF_87 | PF03552.9 | Cellulose synthase | - |
| GF_17 | PF05970.9 | PIF1-like helicase | - |
| GF_11704 | PF00076.17 | RNA recognition motif. (a.k.a. RRM, RBD, or RNP domain) | - |
| GF_12558 | PF03080.10 | Domain of unknown function (DUF239) | - |
| GF_12625 | PF00672.20 | HAMP domain | - |
| GF_947 | PF00321.12 | Plant thionin | - |
| GF_1769 | PF00117.23 | Glutamine amidotransferase class-I | - |
| GF_2760 | PF00646.28 | F-box domain | - |
| GF_321 | PF07690.11 | Major Facilitator Superfamily | - |
| GF_2266 | PF07734.8 | F-box associated | - |
| GF_421 | PF02362.16 | B3 DNA binding domain | - |
| GF_1238 | PF03145.11 | Seven in absentia protein family | - |
| GF_1960 | PF02469.17 | Fasciclin domain | - |
| GF_652 | PF03478.13 | Protein of unknown function (DUF295) | - |
| GF_4385 | PF10551.4 | MULE transposase domain | - |
| GF_3789 | PF00646.28 | F-box domain | - |
| GF_10441 | PF02994.9 | L1 transposable element | - |
| GF_2497 | PF00076.17 | RNA recognition motif. (a.k.a. RRM, RBD, or RNP domain) | - |
| GF_73 | PF03478.13 | Protein of unknown function (DUF295) | - |
| GF_1079 | PF03140.10 | Plant protein of unknown function | - |
| GF_12779 | PF02892.10 | BED zinc finger | - |
| GF_2245 | PF00078.22 | Reverse transcriptase (RNA-dependent DNA polymerase) | - |
| GF_718 | PF00067.17 | Cytochrome P450 | - |
| GF_13796 | PF00106.20 | short chain dehydrogenase | - |
| GF_817 | PF10551.4 | MULE transposase domain | - |
| GF_13311 | PF07816.6 | Protein of unknown function (DUF1645) | - |
| GF_12817 | PF05498.6 | Rapid ALkalinization Factor (RALF) | - |
| GF_230 | PF00125.19 | Core histone H2A/H2B/H3/H4 | - |
| GF_28 | PF03372.18 | Endonuclease/Exonuclease/phosphatase family | - |
| GF_12296 | PF00319.13 | SRF-type transcription factor (DNA-binding and dimerisation domain) | - |
| GF_263 | PF00646.28 | F-box domain | - |
| GF_323 | PF02458.10 | Transferase family | - |
| GF_1404 | PF03552.9 | Cellulose synthase | - |
| GF_11597 | PF00097.20 | Zinc finger, C3HC4 type (RING finger) | - |
| GF_147 | PF00078.22 | Reverse transcriptase (RNA-dependent DNA polymerase) | - |
| GF_46 | PF14111.1 | Domain of unknown function (DUF4283) | - |
| GF_8609 | PF00917.21 | MATH domain | - |
| GF_3627 | PF00179.21 | Ubiquitin-conjugating enzyme | - |
| GF_13386 | PF07168.6 | Ureide permease | - |
| GF_13392 | PF07734.8 | F-box associated | - |
| GF_1540 | PF00407.14 | Pathogenesis-related protein Bet v I family | - |
| GF_3381 | PF00646.28 | F-box domain | - |
| GF_1507 | PF00234.17 | Protease inhibitor/seed storage/LTP family | - |
| GF_13369 | PF06888.7 | Putative Phosphatase | - |
| GF_521 | PF12796.2 | Ankyrin repeats (3 copies) | - |
| GF_11595 | PF00307.26 | Calponin homology (CH) domain | - |
| GF_7852 | PF01453.19 | D-mannose binding lectin | - |
| GF_793 | PF05577.7 | Serine carboxypeptidase S28 | - |
| GF_1819 | PF03018.9 | Dirigent-like protein | - |
| GF_5164 | PF05432.6 | Bone sialoprotein II (BSP-II) | - |
| GF_1023 | PF14227.1 | gag-polypeptide of LTR copia-type | - |
| GF_847 | PF03080.10 | Domain of unknown function (DUF239) | - |
| GF_182 | PF02519.9 | Auxin responsive protein | - |
| GF_13348 | PF01610.12 | Transposase | - |
| GF_8588 | PF02362.16 | B3 DNA binding domain | - |
| GF_7179 | PF00847.15 | AP2 domain | - |
| GF_152 | PF03372.18 | Endonuclease/Exonuclease/phosphatase family | - |
| GF_82 | PF14543.1 | Xylanase inhibitor N-terminal | - |
| GF_373 | PF14299.1 | Phloem protein 2 | - |
| GF_827 | PF13561.1 | Enoyl-(Acyl carrier protein) reductase | - |
| GF_89 | PF14226.1 | non-haem dioxygenase in morphine synthesis N-terminal | - |
| GF_472 | PF01117.15 | Aerolysin toxin | - |
| GF_488 | PF02458.10 | Transferase family | - |
| GF_276 | PF07714.12 | Protein tyrosine kinase | - |
| GF_5204 | PF13966.1 | zinc-binding in reverse transcriptase | - |
| GF_36 | PF00012.15 | Hsp70 protein | - |
| GF_13410 | PF01554.13 | MatE | - |
| GF_803 | PF03018.9 | Dirigent-like protein | - |
| GF_203 | PF01061.19 | ABC-2 type transporter | - |
| GF_12283 | PF00646.28 | F-box domain | - |
| GF_235 | PF13641.1 | Glycosyltransferase like family 2 | - |
| GF_1078 | PF02987.11 | Late embryogenesis abundant protein | - |
| GF_995 | PF05617.6 | Prolamin-like | - |
| GF_301 | PF00240.18 | Ubiquitin family | - |
| GF_818 | PF00141.18 | Peroxidase | - |
| GF_7294 | PF00646.28 | F-box domain | - |
| GF_4275 | PF00319.13 | SRF-type transcription factor (DNA-binding and dimerisation domain) | - |
| GF_2833 | PF08268.7 | F-box associated domain | - |
| GF_672 | PF14244.1 | gag-polypeptide of LTR copia-type | - |
| GF_896 | PF07734.8 | F-box associated | - |
| GF_39 | PF03552.9 | Cellulose synthase | - |
| GF_6202 | PF05938.6 | Plant self-incompatibility protein S1 | - |
| GF_950 | PF00101.15 | Ribulose bisphosphate carboxylase, small chain | - |
| GF_433 | PF00107.21 | Zinc-binding dehydrogenase | - |
| GF_98 | PF00125.19 | Core histone H2A/H2B/H3/H4 | - |
| GF_2015 | PF14144.1 | Seed dormancy control | - |
| GF_13375 | PF01074.17 | Glycosyl hydrolases family 38 N-terminal domain | - |
| GF_120 | PF14111.1 | Domain of unknown function (DUF4283) | - |
| GF_1117 | PF10551.4 | MULE transposase domain | - |
| GF_7212 | PF05004.8 | Interferon-related developmental regulator (IFRD) | - |
| GF_214 | PF07250.6 | Glyoxal oxidase N-terminus | - |
| GF_44 | PF05327.6 | RNA polymerase I specific transcription initiation factor RRN3 | - |
| GF_12297 | PF00304.15 | Gamma-thionin family | - |
| GF_955 | PF00847.15 | AP2 domain | - |
| GF_1004 | PF00428.14 | 60s Acidic ribosomal protein | - |
| GF_222 | PF00646.28 | F-box domain | - |
| GF_144 | PF00501.23 | AMP-binding enzyme | - |
| GF_1104 | PF00295.12 | Glycosyl hydrolases family 28 | - |
| GF_897 | PF01453.19 | D-mannose binding lectin | - |
| GF_10756 | PF02298.12 | Plastocyanin-like domain | - |
| GF_231 | PF00067.17 | Cytochrome P450 | - |
| GF_587 | PF03016.10 | Exostosin family | - |
| GF_490 | PF00190.17 | Cupin | - |
| GF_13371 | PF00026.18 | Eukaryotic aspartyl protease | - |
| GF_445 | PF00646.28 | F-box domain | - |
| GF_1966 | PF00031.16 | Cystatin domain | - |
| GF_5238 | PF00304.15 | Gamma-thionin family | - |
| GF_79 | PF00999.16 | Sodium/hydrogen exchanger family | - |
| GF_4 | PF00069.20 | Protein kinase domain | - |
| GF_1309 | PF04525.7 | Tubby C 2 | - |
| GF_3597 | PF08137.7 | DVL family | - |
| GF_574 | PF03059.11 | Nicotianamine synthase protein | - |
| GF_13393 | PF03478.13 | Protein of unknown function (DUF295) | - |
| GF_57 | PF01565.18 | FAD binding domain | - |
| GF_776 | PF00141.18 | Peroxidase | - |
| GF_1536 | PF00413.19 | Matrixin | - |
| GF_12278 | PF00646.28 | F-box domain | - |
| GF_426 | PF10551.4 | MULE transposase domain | - |
| GF_74 | PF00067.17 | Cytochrome P450 | - |
| GF_12839 | PF09329.6 | Primase zinc finger | - |
| GF_765 | PF00743.14 | Flavin-binding monooxygenase-like | - |
| GF_1116 | PF08646.5 | Replication factor-A C terminal domain | - |
| GF_10413 | PF00646.28 | F-box domain | - |
| GF_1069 | PF00168.25 | C2 domain | - |
| GF_143 | PF00069.20 | Protein kinase domain | - |
| GF_1812 | PF03004.9 | Plant transposase (Ptta/En/Spm family) | - |
| GF_8619 | PF11926.3 | Domain of unknown function (DUF3444) | - |
| GF_3779 | PF10604.4 | Polyketide cyclase / dehydrase and lipid transport | - |
| GF_12823 | PF01476.15 | LysM domain | - |
| GF_13 | PF00069.20 | Protein kinase domain | - |
| GF_12272 | PF06839.7 | GRF zinc finger | - |
| GF_12800 | PF12796.2 | Ankyrin repeats (3 copies) | - |
| GF_13401 | PF00304.15 | Gamma-thionin family | - |
| GF_3278 | PF03168.8 | Late embryogenesis abundant protein | - |
| GF_6168 | PF00069.20 | Protein kinase domain | - |
| GF_12623 | PF02362.16 | B3 DNA binding domain | - |
| GF_14017 | PF14111.1 | Domain of unknown function (DUF4283) | - |
| GF_26 | PF00497.15 | Bacterial extracellular solute-binding proteins, family 3 | - |
| GF_1212 | PF13966.1 | zinc-binding in reverse transcriptase | - |
| GF_13797 | PF14667.1 | Polysaccharide biosynthesis C-terminal domain | - |
| GF_261 | PF00078.22 | Reverse transcriptase (RNA-dependent DNA polymerase) | - |
| GF_312 | PF07993.7 | Male sterility protein | - |
| GF_13379 | PF00271.26 | Helicase conserved C-terminal domain | - |
| GF_1532 | PF03060.10 | Nitronate monooxygenase | - |
| GF_2011 | PF03478.13 | Protein of unknown function (DUF295) | - |
| GF_337 | PF00067.17 | Cytochrome P450 | - |
| GF_196 | PF00854.16 | POT family | - |
| GF_13373 | PF00141.18 | Peroxidase | - |
| GF_13395 | PF07501.7 | G5 domain | - |
| GF_81 | PF03936.11 | Terpene synthase family, metal binding domain | - |
| GF_7318 | PF00657.17 | GDSL-like Lipase/Acylhydrolase | - |
| GF_100 | PF00067.17 | Cytochrome P450 | - |
| GF_101 | PF00916.15 | Sulfate transporter family | - |
| GF_1199 | PF02214.17 | BTB/POZ domain | - |
| GF_23 | PF07714.12 | Protein tyrosine kinase | - |
| GF_2153 | PF00462.19 | Glutaredoxin | - |
| GF_8100 | PF00847.15 | AP2 domain | - |
| GF_1661 | PF00407.14 | Pathogenesis-related protein Bet v I family | - |
| GF_1872 | PF02891.15 | MIZ/SP-RING zinc finger | - |
| GF_40 | PF00232.13 | Glycosyl hydrolase family 1 | - |
| GF_13387 | PF00646.28 | F-box domain | - |
| GF_29 | PF00078.22 | Reverse transcriptase (RNA-dependent DNA polymerase) | - |
| GF_1525 | PF13837.1 | Myb/SANT-like DNA-binding domain | - |
| GF_10432 | PF03004.9 | Plant transposase (Ptta/En/Spm family) | - |
| GF_1546 | PF00141.18 | Peroxidase | - |
| GF_1535 | PF00294.19 | pfkB family carbohydrate kinase | - |
| GF_12273 | PF02362.16 | B3 DNA binding domain | - |
| GF_10424 | PF02721.9 | Domain of unknown function DUF223 | - |
| GF_1040 | PF00069.20 | Protein kinase domain | - |
| GF_5 | PF00082.17 | Subtilase family | - |
| GF_1815 | PF03055.10 | Retinal pigment epithelial membrane protein | - |
| GF_8 | PF07734.8 | F-box associated | - |
| GF_11295 | PF00270.24 | DEAD/DEAH box helicase | - |
| GF_132 | PF03321.8 | GH3 auxin-responsive promoter | - |
| GF_4504 | PF00704.23 | Glycosyl hydrolases family 18 | - |
| GF_1374 | PF05056.7 | Protein of unknown function (DUF674) | - |
| GF_2112 | PF00439.20 | Bromodomain | - |
| GF_1180 | PF02458.10 | Transferase family | - |
| GF_760 | PF02182.12 | SAD/SRA domain | - |
| GF_12279 | PF12776.2 | Myb/SANT-like DNA-binding domain | - |
| GF_629 | PF04030.9 | D-arabinono-1,4-lactone oxidase | - |
| GF_8550 | PF08387.5 | FBD | - |
| GF_899 | PF14244.1 | gag-polypeptide of LTR copia-type | - |
| GF_13400 | PF00646.28 | F-box domain | - |
| GF_128 | PF00083.19 | Sugar (and other) transporter | - |
| GF_13793 | PF07690.11 | Major Facilitator Superfamily | - |
| GF_1254 | PF03439.8 | Early transcription elongation factor of RNA pol II, NGN section | - |
| GF_640 | PF01397.16 | Terpene synthase, N-terminal domain | - |
| GF_509 | PF00009.22 | Elongation factor Tu GTP binding domain | - |
| GF_413 | PF00067.17 | Cytochrome P450 | - |
| GF_13378 | PF10551.4 | MULE transposase domain | - |
| GF_5084 | PF00069.20 | Protein kinase domain | - |
| GF_1563 | PF01596.12 | O-methyltransferase | - |
| GF_414 | PF00150.13 | Cellulase (glycosyl hydrolase family 5) | - |
| GF_6075 | PF00075.19 | RNase H | - |
| GF_2098 | PF01190.12 | Pollen proteins Ole e I like | - |
| GF_1172 | PF13561.1 | Enoyl-(Acyl carrier protein) reductase | - |
| GF_12816 | PF10604.4 | Polyketide cyclase / dehydrase and lipid transport | - |
| GF_428 | PF00201.13 | UDP-glucoronosyl and UDP-glucosyl transferase | - |
| GF_732 | PF03478.13 | Protein of unknown function (DUF295) | - |
| GF_940 | PF00657.17 | GDSL-like Lipase/Acylhydrolase | - |
| GF_425 | PF14547.1 | Hydrophobic seed protein | - |
| GF_2537 | PF13041.1 | PPR repeat family | - |
| GF_107 | PF03936.11 | Terpene synthase family, metal binding domain | - |
| GF_70 | PF00078.22 | Reverse transcriptase (RNA-dependent DNA polymerase) | - |
| GF_2800 | PF00646.28 | F-box domain | - |
| GF_207 | PF14683.1 | Polysaccharide lyase family 4, domain III | - |
| GF_383 | PF00319.13 | SRF-type transcription factor (DNA-binding and dimerisation domain) | - |
| GF_7314 | PF00203.16 | Ribosomal protein S19 | - |
| GF_2596 | PF00004.24 | ATPase family associated with various cellular activities (AAA) | - |
| GF_286 | PF00931.17 | NB-ARC domain | - |
| GF_514 | PF00201.13 | UDP-glucoronosyl and UDP-glucosyl transferase | - |
| GF_58 | PF00201.13 | UDP-glucoronosyl and UDP-glucosyl transferase | - |
| GF_3902 | PF03004.9 | Plant transposase (Ptta/En/Spm family) | - |
| GF_2544 | PF00067.17 | Cytochrome P450 | - |
| GF_1833 | PF03168.8 | Late embryogenesis abundant protein | - |
| GF_1868 | PF03007.11 | Wax ester synthase-like Acyl-CoA acyltransferase domain | - |
| GF_1005 | PF13839.1 | GDSL/SGNH-like Acyl-Esterase family found in Pmr5 and Cas1p | - |
| GF_3238 | PF00569.12 | Zinc finger, ZZ type | - |
| GF_1002 | PF03087.9 | Arabidopsis protein of unknown function | - |
| GF_11607 | PF13207.1 | AAA domain | - |
| GF_8600 | PF05970.9 | PIF1-like helicase | - |
| GF_850 | PF14244.1 | gag-polypeptide of LTR copia-type | - |
| GF_347 | PF02458.10 | Transferase family | - |
| GF_1385 | PF11250.3 | Protein of unknown function (DUF3049) | - |
| GF_8633 | PF07714.12 | Protein tyrosine kinase | - |
| GF_105 | PF00931.17 | NB-ARC domain | - |
| GF_134 | PF02171.12 | Piwi domain | - |
| GF_3524 | PF14244.1 | gag-polypeptide of LTR copia-type | - |
| GF_5333 | PF00646.28 | F-box domain | - |
| GF_1324 | PF13921.1 | Myb-like DNA-binding domain | - |
| GF_11602 | PF12776.2 | Myb/SANT-like DNA-binding domain | - |
| GF_1653 | PF10536.4 | Plant mobile domain | - |

| GF_13047 | PF00332.13 | Glycosyl hydrolases family 17 | + |
| --- | --- | --- | --- |
| GF_112 | PF00305.14 | Lipoxygenase | + |
| GF_8362 | PF13855.1 | Leucine rich repeat | + |
| GF_697 | PF14223.1 | gag-polypeptide of LTR copia-type | + |
| GF_236 | PF00407.14 | Pathogenesis-related protein Bet v I family | + |
| GF_726 | PF00685.22 | Sulfotransferase domain | + |
| GF_67 | PF00702.21 | haloacid dehalogenase-like hydrolase | + |
| GF_90 | PF00067.17 | Cytochrome P450 | + |
| GF_9 | PF00078.22 | Reverse transcriptase (RNA-dependent DNA polymerase) | + |
| GF_1415 | PF13359.1 | DDE superfamily endonuclease | + |
| GF_7385 | PF13639.1 | Ring finger domain | + |
| GF_167 | PF00069.20 | Protein kinase domain | + |
| GF_678 | PF14543.1 | Xylanase inhibitor N-terminal | + |
| GF_5505 | PF00106.20 | short chain dehydrogenase | + |
| GF_909 | PF04937.10 | Protein of unknown function (DUF 659) | + |
| GF_1850 | PF02458.10 | Transferase family | + |
| GF_536 | PF00067.17 | Cytochrome P450 | + |
| GF_322 | PF00201.13 | UDP-glucoronosyl and UDP-glucosyl transferase | + |
| GF_1442 | PF05904.6 | Plant protein of unknown function (DUF863) | + |
| GF_498 | PF14576.1 | Sieve element occlusion N-terminus | + |
| GF_243 | PF00201.13 | UDP-glucoronosyl and UDP-glucosyl transferase | + |
| GF_650 | PF01370.16 | NAD dependent epimerase/dehydratase family | + |
| GF_1691 | PF10377.4 | Autophagy-related protein 11 | + |
| GF_4597 | PF14244.1 | gag-polypeptide of LTR copia-type | + |
| GF_1248 | PF03195.9 | Protein of unknown function DUF260 | + |
| GF_237 | PF00107.21 | Zinc-binding dehydrogenase | + |
| GF_5083 | PF03171.15 | 2OG-Fe(II) oxygenase superfamily | + |
| GF_711 | PF14372.1 | Domain of unknown function (DUF4413) | + |
| GF_1017 | PF00872.13 | Transposase, Mutator family | + |
| GF_548 | PF00201.13 | UDP-glucoronosyl and UDP-glucosyl transferase | + |
| GF_154 | PF00155.16 | Aminotransferase class I and II | + |
| GF_3759 | PF14244.1 | gag-polypeptide of LTR copia-type | + |
| GF_238 | PF00891.13 | O-methyltransferase | + |
| GF_362 | PF13561.1 | Enoyl-(Acyl carrier protein) reductase | + |
| GF_713 | PF13968.1 | Domain of unknown function (DUF4220) | + |
| GF_3 | PF03732.12 | Retrotransposon gag protein | + |
| GF_13622 | PF01466.14 | Skp1 family, dimerisation domain | + |
| GF_3378 | PF13359.1 | DDE superfamily endonuclease | + |
| GF_11973 | PF10377.4 | Autophagy-related protein 11 | + |
| GF_2557 | PF14223.1 | gag-polypeptide of LTR copia-type | + |
| GF_1357 | PF01167.13 | Tub family | + |
| GF_14293 | PF08242.7 | Methyltransferase domain | + |
| GF_56 | PF00190.17 | Cupin | + |
| GF_717 | PF14368.1 | Probable lipid transfer | + |
| GF_241 | PF12609.3 | Wound-induced protein | + |
| GF_1307 | PF00010.21 | Helix-loop-helix DNA-binding domain | + |
| GF_1718 | PF03171.15 | 2OG-Fe(II) oxygenase superfamily | + |
| GF_13043 | PF12214.3 | Cell cycle regulated microtubule associated protein | + |
| GF_1 | PF14432.1 | DYW family of nucleic acid deaminases | + |
| GF_91 | PF02536.9 | mTERF | + |
| GF_1063 | PF00657.17 | GDSL-like Lipase/Acylhydrolase | + |
| GF_1620 | PF03514.9 | GRAS domain family | + |
| GF_50 | PF00067.17 | Cytochrome P450 | + |
| GF_393 | PF00201.13 | UDP-glucoronosyl and UDP-glucosyl transferase | + |
| GF_485 | PF00195.14 | Chalcone and stilbene synthases, N-terminal domain | + |
| GF_8831 | PF04554.8 | Extensin-like region | + |
| GF_161 | PF01095.14 | Pectinesterase | + |
| GF_412 | PF00295.12 | Glycosyl hydrolases family 28 | + |
| GF_804 | PF01425.16 | Amidase | + |
| GF_6212 | PF01476.15 | LysM domain | + |
| GF_24 | PF00069.20 | Protein kinase domain | + |
| GF_170 | PF13086.1 | AAA domain | + |
| GF_1287 | PF00069.20 | Protein kinase domain | + |
| GF_685 | PF00182.14 | Chitinase class I | + |
| GF_3139 | PF05056.7 | Protein of unknown function (DUF674) | + |
| GF_11976 | PF00168.25 | C2 domain | + |
| GF_442 | PF00067.17 | Cytochrome P450 | + |
| GF_10174 | PF07333.7 | S locus-related glycoprotein 1 binding pollen coat protein (SLR1-BP) | + |
| GF_338 | PF01734.17 | Patatin-like phospholipase | + |
| GF_2040 | PF13499.1 | EF-hand domain pair | + |
| GF_357 | PF03018.9 | Dirigent-like protein | + |
| GF_8352 | PF14368.1 | Probable lipid transfer | + |
| GF_567 | PF14223.1 | gag-polypeptide of LTR copia-type | + |
| GF_945 | PF06880.6 | Protein of unknown function (DUF1262) | + |
| GF_751 | PF00504.16 | Chlorophyll A-B binding protein | + |
| GF_1101 | PF00403.21 | Heavy-metal-associated domain | + |
| GF_895 | PF00847.15 | AP2 domain | + |
| GF_1253 | PF03140.10 | Plant protein of unknown function | + |
| GF_585 | PF00657.17 | GDSL-like Lipase/Acylhydrolase | + |
| GF_976 | PF04780.7 | Protein of unknown function (DUF629) | + |
| GF_174 | PF10377.4 | Autophagy-related protein 11 | + |
| GF_11698 | PF14214.1 | Helitron helicase-like domain at N-terminus | + |
| GF_210 | PF00011.16 | Hsp20/alpha crystallin family | + |
| GF_5723 | PF00232.13 | Glycosyl hydrolase family 1 | + |
| GF_287 | PF00854.16 | POT family | + |
| GF_504 | PF00112.18 | Papain family cysteine protease | + |
| GF_6498 | PF08268.7 | F-box associated domain | + |
| GF_5793 | PF05938.6 | Plant self-incompatibility protein S1 | + |
| GF_7513 | PF07727.9 | Reverse transcriptase (RNA-dependent DNA polymerase) | + |
| GF_1696 | PF12796.2 | Ankyrin repeats (3 copies) | + |
| GF_283 | PF00724.15 | NADH:flavin oxidoreductase / NADH oxidase family | + |
| GF_309 | PF00043.20 | Glutathione S-transferase, C-terminal domain | + |
| GF_13619 | PF07714.12 | Protein tyrosine kinase | + |
| GF_480 | PF01490.13 | Transmembrane amino acid transporter protein | + |
| GF_11523 | PF07727.9 | Reverse transcriptase (RNA-dependent DNA polymerase) | + |
| GF_99 | PF00069.20 | Protein kinase domain | + |
| GF_1043 | PF02298.12 | Plastocyanin-like domain | + |
| GF_4413 | PF02362.16 | B3 DNA binding domain | + |
| GF_423 | PF00201.13 | UDP-glucoronosyl and UDP-glucosyl transferase | + |
| GF_562 | PF00891.13 | O-methyltransferase | + |
| GF_596 | PF04043.10 | Plant invertase/pectin methylesterase inhibitor | + |
| GF_1188 | PF05938.6 | Plant self-incompatibility protein S1 | + |
| GF_584 | PF00201.13 | UDP-glucoronosyl and UDP-glucosyl transferase | + |
| GF_345 | PF00067.17 | Cytochrome P450 | + |
| GF_989 | PF02536.9 | mTERF | + |
| GF_1074 | PF13923.1 | Zinc finger, C3HC4 type (RING finger) | + |
| GF_6757 | PF00892.15 | EamA-like transporter family | + |
| GF_11889 | PF14244.1 | gag-polypeptide of LTR copia-type | + |
| GF_12513 | PF04053.9 | Coatomer WD associated region | + |
| GF_559 | PF02458.10 | Transferase family | + |
| GF_213 | PF03080.10 | Domain of unknown function (DUF239) | + |
| GF_11198 | PF00190.17 | Cupin | + |
| GF_478 | PF00043.20 | Glutathione S-transferase, C-terminal domain | + |
| GF_94 | PF13249.1 | Prenyltransferase-like | + |
| GF_9609 | PF00078.22 | Reverse transcriptase (RNA-dependent DNA polymerase) | + |
| GF_1822 | PF01612.15 | 3'-5' exonuclease | + |
| GF_5358 | PF05938.6 | Plant self-incompatibility protein S1 | + |
| GF_216 | PF00201.13 | UDP-glucoronosyl and UDP-glucosyl transferase | + |
| GF_318 | PF00462.19 | Glutaredoxin | + |
| GF_4194 | PF00010.21 | Helix-loop-helix DNA-binding domain | + |
| GF_6017 | PF00847.15 | AP2 domain | + |
| GF_775 | PF00295.12 | Glycosyl hydrolases family 28 | + |
| GF_103 | PF00069.20 | Protein kinase domain | + |
| GF_2305 | PF00657.17 | GDSL-like Lipase/Acylhydrolase | + |
| GF_327 | PF00112.18 | Papain family cysteine protease | + |
| GF_41 | PF07714.12 | Protein tyrosine kinase | + |
| GF_4018 | PF00931.17 | NB-ARC domain | + |
| GF_11 | PF07714.12 | Protein tyrosine kinase | + |
| GF_138 | PF00201.13 | UDP-glucoronosyl and UDP-glucosyl transferase | + |
| GF_1392 | PF03007.11 | Wax ester synthase-like Acyl-CoA acyltransferase domain | + |
| GF_404 | PF02773.11 | S-adenosylmethionine synthetase, C-terminal domain | + |
| GF_783 | PF05496.7 | Holliday junction DNA helicase ruvB N-terminus | + |
| GF_141 | PF00450.17 | Serine carboxypeptidase | + |
| GF_3464 | PF01764.20 | Lipase (class 3) | + |
| GF_1098 | PF00076.17 | RNA recognition motif. (a.k.a. RRM, RBD, or RNP domain) | + |
| GF_304 | PF10551.4 | MULE transposase domain | + |
| GF_12232 | PF00280.13 | Potato inhibitor I family | + |
| GF_1237 | PF13499.1 | EF-hand domain pair | + |
| GF_2654 | PF14244.1 | gag-polypeptide of LTR copia-type | + |
| GF_13627 | PF05938.6 | Plant self-incompatibility protein S1 | + |
| GF_1026 | PF05536.6 | Neurochondrin | + |
| GF_951 | PF00201.13 | UDP-glucoronosyl and UDP-glucosyl transferase | + |
| GF_1135 | PF03140.10 | Plant protein of unknown function | + |
| GF_506 | PF00106.20 | short chain dehydrogenase | + |
| GF_314 | PF03405.9 | Fatty acid desaturase | + |
| GF_11975 | PF13359.1 | DDE superfamily endonuclease | + |
| GF_2111 | PF00385.19 | Chromo (CHRromatin Organisation MOdifier) domain | + |
| GF_1302 | PF01190.12 | Pollen proteins Ole e I like | + |
| GF_1564 | PF00134.18 | Cyclin, N-terminal domain | + |
| GF_10 | PF07714.12 | Protein tyrosine kinase | + |
| GF_882 | PF03081.10 | Exo70 exocyst complex subunit | + |
| GF_876 | PF00141.18 | Peroxidase | + |
| GF_523 | PF07859.8 | alpha/beta hydrolase fold | + |
| GF_2084 | PF05553.6 | Cotton fibre expressed protein | + |
| GF_273 | PF00657.17 | GDSL-like Lipase/Acylhydrolase | + |
| GF_335 | PF00201.13 | UDP-glucoronosyl and UDP-glucosyl transferase | + |
| GF_384 | PF00069.20 | Protein kinase domain | + |
| GF_1109 | PF05641.7 | Agenet domain | + |
| GF_13039 | PF00403.21 | Heavy-metal-associated domain | + |
| GF_1616 | PF07727.9 | Reverse transcriptase (RNA-dependent DNA polymerase) | + |
| GF_13986 | PF12796.2 | Ankyrin repeats (3 copies) | + |
| GF_7460 | PF07714.12 | Protein tyrosine kinase | + |
| GF_184 | PF00067.17 | Cytochrome P450 | + |
| GF_171 | PF02362.16 | B3 DNA binding domain | + |
| GF_716 | PF07734.8 | F-box associated | + |
| GF_146 | PF00022.14 | Actin | + |
| GF_661 | PF04674.7 | Phosphate-induced protein 1 conserved region | + |
| GF_16 | PF00078.22 | Reverse transcriptase (RNA-dependent DNA polymerase) | + |
| GF_1390 | PF00931.17 | NB-ARC domain | + |
| GF_900 | PF00197.13 | Trypsin and protease inhibitor | + |
| GF_86 | PF00069.20 | Protein kinase domain | + |
| GF_435 | PF01545.16 | Cation efflux family | + |
| GF_247 | PF13921.1 | Myb-like DNA-binding domain | + |
| GF_12516 | PF09117.5 | MiAMP1 | + |
| GF_11735 | PF04434.12 | SWIM zinc finger | + |
| GF_4982 | PF00854.16 | POT family | + |
| GF_8575 | PF00931.17 | NB-ARC domain | + |
| GF_346 | PF03087.9 | Arabidopsis protein of unknown function | + |
| GF_8806 | PF13041.1 | PPR repeat family | + |
| GF_35 | PF02705.11 | K+ potassium transporter | + |
| GF_7925 | PF00010.21 | Helix-loop-helix DNA-binding domain | + |
| GF_1131 | PF12796.2 | Ankyrin repeats (3 copies) | + |
| GF_468 | PF00011.16 | Hsp20/alpha crystallin family | + |
| GF_253 | PF00924.13 | Mechanosensitive ion channel | + |
| GF_350 | PF00504.16 | Chlorophyll A-B binding protein | + |
| GF_150 | PF03169.10 | OPT oligopeptide transporter protein | + |
| GF_48 | PF02519.9 | Auxin responsive protein | + |
| GF_449 | PF12697.2 | Alpha/beta hydrolase family | + |
| GF_122 | PF00854.16 | POT family | + |
| GF_6409 | PF05929.6 | Phage capsid scaffolding protein (GPO) serine peptidase | + |
| GF_119 | PF00069.20 | Protein kinase domain | + |
| GF_1425 | PF00931.17 | NB-ARC domain | + |
| GF_1405 | PF04885.8 | Stigma-specific protein, Stig1 | + |
| GF_72 | PF00665.21 | Integrase core domain | + |
| GF_6218 | PF14543.1 | Xylanase inhibitor N-terminal | + |
| GF_63 | PF00067.17 | Cytochrome P450 | + |
| GF_2829 | PF00264.15 | Common central domain of tyrosinase | + |
| GF_954 | PF00190.17 | Cupin | + |
| GF_627 | PF00043.20 | Glutathione S-transferase, C-terminal domain | + |
| GF_1373 | PF04520.8 | Senescence regulator | + |
| GF_398 | PF13962.1 | Domain of unknown function | + |
| GF_1711 | PF00403.21 | Heavy-metal-associated domain | + |
| GF_31 | PF00069.20 | Protein kinase domain | + |
| GF_520 | PF00201.13 | UDP-glucoronosyl and UDP-glucosyl transferase | + |
| GF_1060 | PF00270.24 | DEAD/DEAH box helicase | + |
| GF_10416 | PF14111.1 | Domain of unknown function (DUF4283) | + |
| GF_71 | PF13855.1 | Leucine rich repeat | + |
| GF_1130 | PF03330.13 | Rare lipoprotein A (RlpA)-like double-psi beta-barrel | + |
| GF_516 | PF00280.13 | Potato inhibitor I family | + |
| GF_727 | PF05699.9 | hAT family C-terminal dimerisation region | + |
| GF_14 | PF02992.9 | Transposase family tnp2 | + |
| GF_374 | PF13962.1 | Domain of unknown function | + |
| GF_295 | PF07727.9 | Reverse transcriptase (RNA-dependent DNA polymerase) | + |
| GF_6969 | PF00665.21 | Integrase core domain | + |
| GF_3756 | PF03168.8 | Late embryogenesis abundant protein | + |
| GF_1545 | PF13962.1 | Domain of unknown function | + |
| GF_1469 | PF14215.1 | bHLH-MYC and R2R3-MYB transcription factors N-terminal | + |
| GF_4165 | PF00665.21 | Integrase core domain | + |
| GF_532 | PF00931.17 | NB-ARC domain | + |
| GF_211 | PF03124.9 | EXS family | + |
| GF_2056 | PF04937.10 | Protein of unknown function (DUF 659) | + |
| GF_153 | PF00544.14 | Pectate lyase | + |
| GF_10372 | PF00069.20 | Protein kinase domain | + |
| GF_1042 | PF10551.4 | MULE transposase domain | + |
| GF_406 | PF14543.1 | Xylanase inhibitor N-terminal | + |
| GF_10414 | PF03478.13 | Protein of unknown function (DUF295) | + |
| GF_5239 | PF00428.14 | 60s Acidic ribosomal protein | + |
| GF_255 | PF00909.16 | Ammonium Transporter Family | + |
| GF_136 | PF13855.1 | Leucine rich repeat | + |
| GF_76 | PF00069.20 | Protein kinase domain | + |
| GF_352 | PF01699.19 | Sodium/calcium exchanger protein | + |
| GF_13632 | PF03754.8 | Domain of unknown function (DUF313) | + |
| GF_1353 | PF00069.20 | Protein kinase domain | + |
| GF_359 | PF00407.14 | Pathogenesis-related protein Bet v I family | + |
| GF_1178 | PF02767.11 | DNA polymerase III beta subunit, central domain | + |
| GF_355 | PF06888.7 | Putative Phosphatase | + |

**Supplementary Information Table S9.** The identified terpene synthase genes identified in *Akebia trifoliata* subsp. *australis* genome.

EVM0013329.1

EVM0016323.1

EVM0025552.1

EVM0012830.1

EVM0008645.1

EVM0021346.1

EVM0002447.1

EVM0018724.1

EVM0020621.1

EVM0001040.1

EVM0021615.1

EVM0000266.1

EVM0015000.1

EVM0016397.1

EVM0010491.1

EVM0015211.1

EVM0011074.1

EVM0010964.1

EVM0009661.1

EVM0008909.1

EVM0020802.1

EVM0024097.1

EVM0023226.1

EVM0016004.1

EVM0000942.1

EVM0007537.1

EVM0016604.1

EVM0004144.1

EVM0001043.1

EVM0014977.2

EVM0005270.1

EVM0008755.1

EVM0004724.1

**Supplementary Information Table S10.** Significantly enriched KEGG pathways of correlations in *Akebia trifoliata* subsp. *australis* seeds from different developmental periods

| Pathway | FDR | Pathway ID |
| --- | --- | --- |
| May-vs-June |  |  |
| Plant hormone signal transduction | 1.49E-06 | ko04075 |
| ABC transporters | 1.24E-03 | ko02010 |
| Nitrogen metabolism | 2.03E-02 | ko00910 |
| Starch and sucrose metabolism | 2.48E-02 | ko00500 |
| Metabolic pathways | 2.75E-02 | ko01100 |
| Brassinosteroid biosynthesis | 3.86E-02 | ko00905 |
| Biosynthesis of secondary metabolites | 4.59E-02 | ko01110 |
| June-vs-Junly |  |  |
| Ribosome | 1.34E-09 | ko03010 |
| DNA replication | 1.48E-03 | ko03030 |
| Photosynthesis | 1.58E-03 | ko00195 |
| ABC transporters | 3.85E-02 | ko02010 |
| Flavonoid biosynthesis | 3.85E-02 | ko00941 |
| July-vs-Aug |  |  |
| Photosynthesis | 8.83E-08 | ko00195 |
| Photosynthesis - antenna proteins | 8.83E-08 | ko00196 |
| Biosynthesis of secondary metabolites | 2.65E-05 | ko01110 |
| Plant-pathogen interaction | 2.54E-03 | ko04626 |
| Metabolic pathways | 6.83E-03 | ko01100 |
| Indole alkaloid biosynthesis | 1.24E-02 | ko00901 |
| Phenylpropanoid biosynthesis | 2.07E-02 | ko00940 |
| ABC transporters | 3.42E-02 | ko02010 |
| Aug-vs-Sep |  |  |
| Biosynthesis of secondary metabolites | 1.17E-06 | ko01110 |
| Glutathione metabolism | 4.54E-04 | ko00480 |
| Metabolic pathways | 1.18E-03 | ko01100 |
| Linoleic acid metabolism | 2.00E-02 | ko00591 |
| alpha-Linolenic acid metabolism | 2.52E-02 | ko00592 |
| Plant hormone signal transduction | 3.30E-02 | ko04075 |
| Tyrosine metabolism | 3.92E-02 | ko00350 |
| Zeatin biosynthesis | 3.92E-02 | ko00908 |
| Indole alkaloid biosynthesis | 3.92E-02 | ko00901 |
| Glycerolipid metabolism | 3.92E-02 | ko00561 |
| Phenylpropanoid biosynthesis | 4.03E-02 | ko00940 |
| Carotenoid biosynthesis | 4.25E-02 | ko00906 |

**Supplementary Information Table S11.** The identified *FAD* genes identified in *Akebia trifoliata* subsp. *australis* genome.

>EVM0000750.1|FA_desaturase

MGAGGRMPVPTTKKEVLDRVPYSKPPFTLSEIKKAIPPHCFHRSILHSFSYVFIDLTIAFLFYYSTTYFPLLPHPLSVLTWPIYWILQGCLLTGVWVIAHECGHHAFSDYQWLDDTVGFILHSFLLVPYFSWKYSHRRHHSNTACLDKDEVFVPKPKSKIPWFSKYLNNPPGRVLTLTTTLLLGWPLYLMFNVSGRHYDQFASHFNPNAPIYSDRERLQIYLSDAGILAVSYGLFCLCTLKGLSWVLCVYGVPLLIVNGFLVLITFLQHTHPSLPHYDSSEWDWLRGALATVDRDYGVLNKVFHNITDTHVAHHLCSTMPHYHAMEATKVIKPILGEYYQFDGTPFYKAMWREARECLYVEPDDENNKGVFWYRNKFDN

>EVM0000977.1|FA_desaturase

MASWVLSECGLRPLPHVFPRPRTGIISNKNPNTKIRLPSCKIGGGVRGTDLKPIWASIGSRNRDLGLKVSAPSRLVSVDEEDREGESINGVNGIGNEEKFNPGAPPPFGLADIRAAIPKHCWVKDAWRSMSYVVRDTVVVFGLAAAAAYLNNWAVWPLYWAAQGTMFWALFVLGHDCGHGSFSSNHKLNSVVGHLLHSSILVPYHGWRISHRTHHQNHGHVENDESWHPLSEKTYRGLDVITRMLRFTVPFPMLAYPFYLWSRSPGKKGSHFHPNSDLFVPKERKDVITSTVCWTAMLALIVGLSCVMGPVQMLKLYGIPYWIFVMWLDFVTYLHHHGHEDKLPWYRGKEWSYLRGGLTTLDRDFGLINNIHHDIGTHVIHHLFPQIPHYHLVEATEAAKPVLGKYYREPKKSGPLPFHLIGTLISSMRQDHYVSDTGEILYYQTDLQHGSSQIKSE

>EVM0001669.1|FA_desaturase

MACGLADLRFNFMGSHQIPIRTSKFPTQHSQGICYLKLGSFIRRGIKHHRCLSHQKKTQIIKAVAVPVQLSALDSAEHRKKLSESYGFTKIGEPLPENITMRDITDTLPKKVFVIDDVKAWKSVLISISSYALGIFMISKAPWYLLPLAWAWTGTAVTGFFVIGHDCAHKSFSRNKLVEDIVGTLAFLPLIYPYEPWRFKHDRHHAKTNMLSEDTAWLPVLRKEFDSSPVFRKAIIFGYGPFRTWMSISHWVIQHFNLEKFRPNEVTRVKISLACVFAFMAIGWPLIIYKTGVMGWIKFWLMPWLGYHFWMSTFTMVHHTAPHIPFKSSEEWNAAQAQLNGTVHCDYPRWIEILCHDINVHIPHHISPRIPSYNLRAAHQSLQENWGKYLNEAKWNWRLMKTILTMCHVYSEEHNYVAFDEIAPEDSQPITFLRTVMPDYA

>EVM0002925.1|FA_desaturase

MALKLGPLTFQSNKYPCFGVPLAGNLISPKVSMSSNLRSTSSKEFGYFKKPLDTSRDVRVLVNHCMSPQTVEIFNSLDDWAKNNILIHLKPVENCWQPQNFLPNPASEGFYEQVEELRERMKEIPDDYFIVLVGDMITEEALPTYQTMLNTMDGMRDKTENRHGDLLNKYLYLSGRVDMRQIEKTIQYLIGSGMDFRAESGTYHGLIYTSFQERATFVSHGNTAKLAKEHGDFILAQICGTIAADEKRHETAYTKIVAKLFEIDEDGTMLAFADMMKHKIVMPAQLMYDGKDDNLFKNFSAVAQRLGVYTANDYANILEFFVGRWNVEKLTGLSGEGRKAQDYVCSLASKVRRLEESSARRAKQAPTIPFSWIFDREV

>EVM0003023.1|FA_desaturase

MLKIVTDTYKGILIILTESKIVFKHQTLSKVLLTVLFRICIQTHNSYPPTPDSTSKSTYESTNNLKKPFSPPHKVHVQVTHSMPPRKVEIFKSLEDWAENNILVHLKPVEKCWQPHDFLPDSASDGFYEQVKELRERAKEIPDDYFVVLVGDMITEEALPTYQTFLNCLDGVRDETGASLTSWAIWTRAWTAEENRHGDLLNKYLYLSGRVDMKQIEKTIQYLIGSGMDVGTENNPYLGFIYTSFQERATFISHGNTARLAKDYGDFKLAQICGTIGADEKRHETAYTKIVKKLFEIDPNGTILAFSDMMKKRILMPAYLMYDGRDDDLFERFSATAKRLGVYTAKDYANIVEFFVGQWNVEKLTGLSGESRKAQDYICGLAPKFRKLEERAFERAKQAPIVPCSWILDREVKL

>EVM0006409.1|FA_desaturase

MGAGGRMSVPPTKEKHEVLGRVPTSKPPFTLSQLKKAIPPRCFERSVLRSFSYVIIDLVIVFLFYYIATTFFPLLPQPVSFIAWPIYWACQGSVLTGVWVIAHECGHHAFSDYQWLDDTVGLVLHSCLLVPYFSWKYSHRRHHSNTGSLERDEVFVPKPKSKVSWFSKYLNNPPGRVLTLAITLLLGWPLYLAFNVSGRPYDRFACHFDPYGPIYNDRERKQIYLSDAGIVAVTYGLIRLCAAKGLSWVFCVYGVPLLIVNGFLVLITFLQHTHPSLPHYDSSEWDWLKGALATVDRDYGVLNKVFHNITDTHVAHHLFSTMPHYNAMEATKAIKPILGEYYQFDETPFYKAMWREARECLYVEPDEDAHDNGVFWYRNKF

>EVM0006852.1|FA_desaturase

MEAKQSKIMFSDVEVKNKRKALDRSKKWNSADAAYVVGMVAVHVLCLFAPFTFNWGAFMAAVTLYVMTGLLGITLCFHRYLTHKSFKLPKVLEYFFAYCGVQAVQGRPMDWVSTQRNHHKFADSDKDPHSPTEGFWHSHINWIFDSNYVKQKRGDVSNVRDLEEQAFYRFLQDTYIAHPIILGALLYAYGGFPYLVWGMGVRIAWVYHITFLVNSACHVWGYQSWNTGDLSKNNWLVALVSFGDGWHNNHHAFEFSARHGLEWWEFDMAYYVVKLIEFFGLASDVKVPSKLQLQKMPLSNGTGGVHKENMFHSNGMKTEVAK

>EVM0007876.1|FA_desaturase

MEKVKNYISSEELKKHTKPGDLWISIQGKVYNVTDWTKDHPGGDLPLMNLAGQDVTDAFVAFHPGSAWKYLDQFFIGYLEDYSVSEVSKDYRRLASEFAKSGLFEKKGHGILLSICCMAFLFSLCVLGVLWSNNVWVHLLSGGLLGVIWMQSGFIGHDSGHYNIMLSPKLNRFMQILTGNCVTGISIGWWKWTHTAHHIAVNSLDFDPDLQHIPFLAVSSTIFNSLTSCFYGRKMVFDSFARFFISYQHWTFYPVMAVARVNLFAQSFMLLLSKRRVPNRGLELLGLCVFWVWFSLLISCLPNWGERVMFVLASFVVSGIQHVQFCLNHFSAHVYVGPPLANDWFEKQTKGSIDISCSTWMDWFHGGLQYQIEHHLFPRLPRCHLRKISPLVRELCKKHTLPYVSVSFLEANKLTIGTLRDAALQARDFTNPIPKNLLWEAVNTHG

>EVM0008796.1|FA_desaturase

MALKLSPLTFQSNKYPCFGAPLAANLISPKVNHCMSPQTAEIFNSMDDWGNNNILIHLKPVENCWQPQNFLPNPASEGFYEQVEELRERVKEISDDYFIVLVGDMITEEALPTYQTMLNTLDGVRDKTENRHGDLLNKYLYLSGRVDMSQIEKAIQYLIGSGMDFRAENSACHGLIYTSFQERATFVSHGNTVKLAKEHGDFILAQICGTIATDEKRHETAYTKIVKKLFKIDEDGTMLAFADMMKHKIVMPTQVMYDGKDDNLFKNFYVVAQRLGVYTANDYANILEFFVGRWNVEKLTGLSGEGRKAQDYVCSLAPKVRILEESSARRAKQAPTIPFSWIFDREV

>EVM0010300.1|FA_desaturase

MALKLSLLTFQSNKYPCFGVPLAGNLTSPKVSMSSNLLSSSSKEFGYFKKPLDTPRDVHVQVTHCMSPQMAEIINSLDDWMKNNILIHLKPVENCWQPQNFLPNPASEGFYEQVEELRERVKEIPDDYFIVLVGDMITEEALPTYQTMLNNKDGVRDKTGCSPSSWAVWTRAWTAEENRHGDLLNKYLYLSGRVDMSQIEKTIQYLIGSGMDFPPESSAYHGLIYTSFQERATFVSHGNTAKLAKEHGDFILAQICGAIAADEKRHETAYTKFVEKLFEIDEDGTMLAFADMMKNKIVMPAQLMYDGKDDNLFKNFSAVAQRLGVYTANDYANILEFFVGRWNVEKLTGLSGEGRKAQDYVCSLAPKVRRLAELSVRRAKQAPTIPFSWIFDREVKL

>EVM0011340.1|FA_desaturase

MALKLSPLTFQSQKLPSLGLPHVSNLRTSKVFMASTLRSTFSEEVDNLKKPCSPPREVHVQVTHSMPPEKIEIFKSLEDWAENNILTHLKPVEKCWQPQDFLPDPSSDGFYDQVKDLRERAKEIPDDYYIALVGDMITEEALPTYQTMLNTLDGVRDETGASLTSWAVWTRAWTAEENRHGDLLNKYLYLCGRVDMKQIEKTIQYLIGSGMDPRTENNPYLGFIYTSFQERATFISHGNTARHAKDYGDLKLAQICGTIAADEKRHETAYTKIVEKLFEIDPDDTIVSFADMMKKKISMPAHLMYDGQDDNLFEHFSAVTQRLGVYTAKDYADILEFLVGRWNLEGLSVSSGEGRKAQDYVCGLAPRIRKLEERAQGRAKKAPIVPFSWIFNKEVQL

>EVM0011897.1|FA_desaturase

MALKLSPLTFQSNKYPCFGVPLAGNLISPKVSMSSNLRSTSSKEFGYFKKPLDTSRDVRVLVNHCMSPQTVEIFNSLDDWAKNNILIHLKPVENCWQPQNFLPNPASEGFYEQVEELRERMKEIPDDYFIVLVGDMITEEALPTYQTMLNTMDGMRDKTENRHGDLLNKYLYLSGRVDMRQIEKTIQYLIGSGMDFRAESGTYHGLIYTSFQERATFVSHGNTAKLAKEHGDFILAQICGTIAADEKRHETAYTKIVAKLFEIDEDGTMLAFADMMKHKIVMPAQLMYDGNDDNLFKNFSAVAQSFLGRWNVEKLTGLSGEGRKAQDYVCSLASKVRRLEESSARRAKQAPTIPFSWIFDREV

>EVM0011941.1|FA_desaturase

MALIAASPNTRFHPSSPIIHPIRSPKQNSISSNPRVFSSISLNPMKQNMKTHFNSFTFTHLRSIKKRWVTVLASSGSAAAENYRKILLSDVAVNPKRRVYWGRKWNFHDIATASIVLAMHILCLFAPFAFNWPAFWVAVSLYIITGLFGITLSFHRNLSHRSFRLPKWLEYFFAYCGVQAAQGNPMDWVSTHRYHHQFVDSDRDPHSPIEGFWYSHTSWLFDTNSIVERCGEPNNVGDLQKQPFYRFLRRTYIVHPIALGVLLYGLGGFPFLVWGMAVRIVWVYHITWLVNSACHVWGHQAWNTGDLSRNNWWVALLAFGEGWHNNHHAFEFSARHGLEWWQLDMTWYIVRLLQVIGLATDVKVPSEIQKERVSISNKNMTIQD

>EVM0012121.1|FA_desaturase

MALKLSPLTFQSNKYPCFGVPLAGNLTSPKVSMSSNLLSSSSKEFGYFKKPLDASRDVHVQVTHCMSPQMAEIFNSLDDWAKNNILIHLKPVENCWQPQNFLPNPTSEGFHEQVEELRERVKEIPDDYFIVLVGDMITEEALPTYQTMLNNIDGMRDKTENRHGDLLNKYLYLSGRVDMSQIEKTIQYLIGSGMDFRAENSACHGLIYTSFQERATFVSHGNTAKLAKEHGDFILAQICGTIAADEKRHETAYTKIVEKLFEIDEDGTMLAFADMMKHKIVMPAQLMYDGKDDNLFKNFSAIAQRLGVYTTNDYANILEFFVGRWNVEKLTGLSGEGRKAQDYVCSLASKVRRLEESNARRAKQTPTIPFSWIFDREV

>EVM0014854.1|FA_desaturase

MALKLSPLTFQSNKYPCFGVPLAGNLTSPKVSMSSNLLSSPSKEFGYFKKPLDASRDVHVQVTHCMSPQMAEIFNSLDDWAKNNILIHLKPVENCWQPQNFLPNPTSEGFHKQVEELRERVKEIPDDYFIVLVGDMITEEALPTYQTMLNNIDGMRDKTGCSPRSWAVWTRAWTAEENRHGDLLNKYLYLSGRVDMSQIEKTIQYLIGSGMDFRAENSACHGLIYTSFQERATFVSHGNTVKLAKEHGDFILAQICGTIAADEKRHETAYTKIVEKLFEIDEDGTMLAFADMMKHKIVMPAQLMYDGKDDNLFKNFSAVAQRLGVYTANDYANILEFFVGRWNVEKLTGLSGEGRKAQDYVCSLASKVRRLEESSARRAKQAPTIPFSWIFDREV

>EVM0015770.1|FA_desaturase

MGVEEREEIGVMATDFFWSYTDEPHASRRRQILSQYPQIRELFGPDPWAFLKITVVVLLQLWTAAFLYNSGWLKILTVAYFFGSFLNHNLFLAIHELSHNLAFSTPIYNRWLGIFANLPIGVPMSVTFQKYHLEHHRFQGVDGIDVDIPSQTEARVVTNIFTKSIWVLLQLFFYALRPLFLNPKPPGLWEFMNFVIQLSLDATMVYFWGWKSLGYMILSTFVGGGMHPMAGHFISEHYIFKPDQETYSYYGPLNLLTWNVGYHNEHHDFPRIAGCKLYKVKEIAPEYYEGFKSYKSWSQVIYMYIMDRTVGPYSRMKRGNVASKSSTTMKKYE

>EVM0016811.1|FA_desaturase

MGAGGRMPVPTTKKEVLDRVPYSKPPFTLSEIKKAIPPHCFHRSILRSFSYVFIDLTIAFLFYYSTTYFPLLPHPLSVLTWPIYWILQGCLLTGVWVIAHECGHHAFSDYQWLDDTVGFILHSFLLVPYFSWKYSHRRHHSNTASLDRDEVFVPKPKSKIPWFSKYLNNPPGRVLTLTTTLLLGWPLYLMFNVSGRHYDQFASHFDPNAPIYSDRERLQIYLSDAGILAVSYGLFCLCTLKGLSWVLCVYGVPLLIVNGFLVLITFLQHTHPSLPHYDSSEWDWLRGALATVDRDYGVLNKVFHNITDTHVAHHLCSAMPHYHAMEATKAIRPILGAYYQFDGTPFYKAMWREARECLYVEPDDENNKGVFWYRNKFDN

>EVM0018447.1|FA_desaturase

MALKLSPLTFQSNKYPCFGVPLAGNLISPKVSMPSNLRSISSKGFGYFKKPLDTSRDVHVLVNHCMSPQMAEIFNSLDDWAKNNILIHLKPVENCWQPQNFLPNPASEGFYEQVKELRERMKEIPDDYFIVLVGDMITEEALPTYQTMLNTMDEMRDKTENRHGDLLNKYLYLSGRVDMRQIEKTIQYLIGSGMDFRVENSVCHGLTYTSFQERATFVSHGNTAKLAKEHGDFILAQICGTIAADEKRHETAYTKIVAKLSEIDEDGTMLAFADMMKHKIVMPAQLMYDGKDDNFFKNFSAVAQRLGVYTANDYANILEFFVGRWNVEKLTGLSGEGRKAQDYVCSLASKIRRLEGSSARRAKQAPTIPFIWIFDREV

>EVM0018971.1|FA_desaturase

MREKWGVEERKEIGMMAMDFLWSYTDKPHASRRQHILSQYPQIRELFGPDPWAFFNIIILTVAYFFDYFLIHNLFLAIHEPSHNFAFSNPVYNRCLKSFANFLIGVPMSVTFQKYHLEHHRFQGVDGIDVDIPSQIEVRVVTNILTKSIWVFLQLFFYIFWPLFLTPQSMGTYSYYGPLNLLTWSVGYHNEHHDFPRIASSKLYKGFKSYKSWSQVIYMYIMDQTI

>EVM0019545.1|FA_desaturase

MALKLSPLTFQSQKYPSFGLPPQANLRSPKVFMASTLRSTSTKEVETLKKPFSPPREVHEQVLHSMPPQKIEIFKSLEDWAEENILVHLKPVEKCWQPQDFLPDPASEGFYDQVKELRERAKEIPDDYFIALVGDMITEEALPTYQTMLNTLDGVRDETGASPTSWAVWTRAWTAEENRHGDLLNKYLYLSGRVDMKQIEKTIQYLIGSGMDPRTENSPYLGFIYTSFQERATFISHGNTGRLAKEHGDFKLAQICGIIAADERRHEIAYTKIVEKLFEIDPNDTILAFADMMKKKISMPAHLMYDGRDDNLFENFSAVAQRLGVYTAKDYADILEFLVKRWKVEKLTGLSGEGRKAQDYVCGLAPRFRRLEERAVGRAKEASVVPFSWIFDREVKL

>EVM0019590.1|FA_desaturase

MQSTILSLYPTQFFSLKKGRNLTHNIRFRSHPVVSAISTLPVRSQVTHSLPPEKLEIFKSLEGWATESVLPLLKPVEDCWQPSDFLPDPTSSSDSFEDEVRALRDRTDRLSDDYFVVLVGDMITEEALPTYQSMINTLDGVRDESGASRSPWANRHGDLLKTYLYLSGRVDMAMVERTVQYLIGAGMEPGTENNPYLGFIYTSFQERATFISHGNTARLAKESGDPTLARICGTIASDEKRHEMAYANIVQKLLEVDPTGAMLAIEDMMRKKITMPAHLMSDGQDPHLFEHFSAVAQRIGVYTAQDYADILEFLVKRWKLEKIEGLTSKGRHAQDFVCGLAPRLRKLQERADERARNMKPHGVRCLKSNSNRRLKLQI

>EVM0021657.1|FA_desaturase

MADQKIIFISTQGLKNHNKPGNLWISIQGKVYNVTDWVKHHPGGALPLLNLSGQDVTNAIVAYHPSSVWKHLDQFFIGYLSDYQISEVSKDYRRLVSEFTKLGLFDKKGHGVYGVLWSNSVFVHLCCGGLMGFIWIQSGWLGHDSGHYQIMSSRGFNRFVQILSGNCLTGISIAWWKWNHNAHHIACNSLEFDPDLQHTPFFVVSSSFFDSLMSYFYKRKMNFDSFTRFLVSYQHWTFYPVMCFARINLYAQSFLLLFSKRRVPNRGQEIVSLLIFWIWYPLIVSSLPNWGERVMFVLASFVVTGIQHVQFCLNHFASSVYIEHHLFPRLPRCHLRKISPFVKELCKKHNLPYISVSFWEANAMTVWTLRTVALQGGDLTNPIPENLLWEAVNTRG

>EVM0022447.1|FA_desaturase

MALNLSLLTFQSNKYPCFGVPLAGNLTSPKVSMSSNLLSSSSKEFGYFKKPLDTPRDVHVQVTYCMSPQMAEIINSLDDWMKNNILIHLKPVENCWQPQNFLPNPASEGFYEQVEELRERVKEIPDDYFIVLVGDMITEEALPTYQTMLNNKDGVRDKTGCSPSSWAVWTRAWTAEENRHGDLLNKYLYLSGRVDMSQIEKTIQYLIGSGMDFPPESSAYHGLIYTSFQERATFVSHGNTAKLAKEHGDFILAQICGAIAADEKCHETAYTKFVEKFFEIDEDGTMLAFADMMKNKIVMPAQLMYDGKDDNLFKNFSVVAQRLGVYTANDYANILEFFVGRWNVEKLTGLSGEGRKAQDYVCGLAPKVRRLAELSVRRAKQAPTIPFSWIFDREVKL

>EVM0022475.1|FA_desaturase

MATWVSSTLSLRSIFRLFPRPRSGFLSKRNGVVCRKEIEVERKEKEEKINGFNGDGEEIDFDPSAPPPFKVGEIRAAIPQHCWIKNPWRSMSYVVKDIAVVLIFAFLAAYFNSWVVWPLYWVGQGTMFWALFVLGHDCGHGSFSNNNILNSVVGHLLHSFILVPYHGWRISHRTHHQNHGHVEKDESWYPLSEKTYKNMDQTSRKLRFTIPFPMLAYPLYLWYRSPGKQGSHFNPNSDLFSPNERKDVITSTLCWTAMVILLVVTSCVVGPIKVLNFYGVPYLIFVMWLDFVTYLHHHGHEQKLPYYRGQEWSYLRGGLTTVDRDYGWINNIHHDIGTHVVHHLFPQIPHYHLIEATKAAKPVLGKYYREPKKSGPLPFHLIGNLVKSMKQDHYVSDTGDIVYYQTDPLLYESSTKKTE

Supplementary Information Table S12. Primers used in this study.

| Gene ID | Primers |
| --- | --- |
| EVM0011340-F | 5' GAAGCCAGTGGAGAAGTGTT 3' |
| EVM0011340-R | 5' CCATCTAGGGTGTTAAGCATT 3' |
| EVM0019545-F | 5' CTACTTTATTGCCTTGGTTGG 3' |
| EVM0019545-R | 5' AGGTCTCCGTGCCTGTTCTC 3' |
| EVM0019590-F | 5' CAGCCGTCCGATTTCTTACC 3' |
| EVM0019590-R | 5' GGCAGTGCCTCTTCCGTGAT 3' |
| EVM0006409-F | 5' GCGACGAAGGCAATCAAACC 3' |
| EVM0006409-R | 5' GCATCCTCATCTGGCTCAAC 3' |
| EVM0000977-F | 5' ATGACGAATCATGGCACCCG 3' |
| EVM0000977-R | 5' AAGGCACCGTAAACCGCAAC 3' |
| EVM0001669-F | 5' AAGACAGGCGTCATGGGTTG 3' |
| EVM0001669-R | 5' TTCCGAAGATTTGAATGGTATGTG 3' |
| EVM0000750-F | 5' TCTCCTCACTGGCGTATGGG 3' |
| EVM0000750-R | 5' ACGACGATGGCTGTATTTCC 3' |
| EVM0022475-F | 5' TTACTTGCATCACCACGGACAC 3' |
| EVM0022475-R | 5' TCAACGGTCGTAAGCCCTCC 3' |
| EVM0009451-F | 5' CTGAAGAACAATATGCCACCAA 3' |
| EVM0009451-R | 5' TAGGGAACACTGTCACAAATGC 3' |
| EVM0021590-F | 5' CAGTGTTGGTTCCTGCCGTTAT 3' |
| EVM0021590-R | 5' TGCTCAATCTTGTCCGCTCC 3' |
| EVM0024046-F | 5' GAAGTTCATAACAGCAGCAA 3' |
| EVM0024046-R | 5' GGTGTAGCAATAATCAGGGT 3' |
| EVM0018287-F | 5' ATGCGTAGAGTGTACCCTGTT 3' |
| EVM0018287-R | 5' TCCCTTGTCCCAACTCCTTA 3' |
| EVM0012715-F | 5' CATTACCATTGCTGCGTTCT 3' |
| EVM0012715-R | 5' CTGTATCTGCTATCCTGCTTC 3' |
| EVM0001370_actin_F | 5' TCAATCCCAAGGCAAACAGA 3' |
| EVM0001370_actin_R | 5' ATCCAGCACAATACCAGTCG 3' |
